# Search for the direct production of charginos and neutralinos in final states with tau leptons in $\sqrt{s} = 13\,\mathrm{TeV}$*pp* collisions with the ATLAS detector

**DOI:** 10.1140/epjc/s10052-018-5583-9

**Published:** 2018-02-22

**Authors:** M. Aaboud, G. Aad, B. Abbott, O. Abdinov, B. Abeloos, S. H. Abidi, O. S. AbouZeid, N. L. Abraham, H. Abramowicz, H. Abreu, R. Abreu, Y. Abulaiti, B. S. Acharya, S. Adachi, L. Adamczyk, J. Adelman, M. Adersberger, T. Adye, A. A. Affolder, Y. Afik, T. Agatonovic-Jovin, C. Agheorghiesei, J. A. Aguilar-Saavedra, S. P. Ahlen, F. Ahmadov, G. Aielli, S. Akatsuka, H. Akerstedt, T. P. A. Åkesson, E. Akilli, A. V. Akimov, G. L. Alberghi, J. Albert, P. Albicocco, M. J. Alconada Verzini, S. C. Alderweireldt, M. Aleksa, I. N. Aleksandrov, C. Alexa, G. Alexander, T. Alexopoulos, M. Alhroob, B. Ali, M. Aliev, G. Alimonti, J. Alison, S. P. Alkire, B. M. M. Allbrooke, B. W. Allen, P. P. Allport, A. Aloisio, A. Alonso, F. Alonso, C. Alpigiani, A. A. Alshehri, M. I. Alstaty, B. Alvarez Gonzalez, D. Álvarez Piqueras, M. G. Alviggi, B. T. Amadio, Y. Amaral Coutinho, C. Amelung, D. Amidei, S. P. Amor Dos Santos, S. Amoroso, G. Amundsen, C. Anastopoulos, L. S. Ancu, N. Andari, T. Andeen, C. F. Anders, J. K. Anders, K. J. Anderson, A. Andreazza, V. Andrei, S. Angelidakis, I. Angelozzi, A. Angerami, A. V. Anisenkov, N. Anjos, A. Annovi, C. Antel, M. Antonelli, A. Antonov, D. J. Antrim, F. Anulli, M. Aoki, L. Aperio Bella, G. Arabidze, Y. Arai, J. P. Araque, V. Araujo Ferraz, A. T. H. Arce, R. E. Ardell, F. A. Arduh, J.-F. Arguin, S. Argyropoulos, M. Arik, A. J. Armbruster, L. J. Armitage, O. Arnaez, H. Arnold, M. Arratia, O. Arslan, A. Artamonov, G. Artoni, S. Artz, S. Asai, N. Asbah, A. Ashkenazi, L. Asquith, K. Assamagan, R. Astalos, M. Atkinson, N. B. Atlay, K. Augsten, G. Avolio, B. Axen, M. K. Ayoub, G. Azuelos, A. E. Baas, M. J. Baca, H. Bachacou, K. Bachas, M. Backes, P. Bagnaia, M. Bahmani, H. Bahrasemani, J. T. Baines, M. Bajic, O. K. Baker, E. M. Baldin, P. Balek, F. Balli, W. K. Balunas, E. Banas, A. Bandyopadhyay, Sw. Banerjee, A. A. E. Bannoura, L. Barak, E. L. Barberio, D. Barberis, M. Barbero, T. Barillari, M-S Barisits, J. T. Barkeloo, T. Barklow, N. Barlow, S. L. Barnes, B. M. Barnett, R. M. Barnett, Z. Barnovska-Blenessy, A. Baroncelli, G. Barone, A. J. Barr, L. Barranco Navarro, F. Barreiro, J. Barreiro Guimarães da Costa, R. Bartoldus, A. E. Barton, P. Bartos, A. Basalaev, A. Bassalat, R. L. Bates, S. J. Batista, J. R. Batley, M. Battaglia, M. Bauce, F. Bauer, H. S. Bawa, J. B. Beacham, M. D. Beattie, T. Beau, P. H. Beauchemin, P. Bechtle, H. P. Beck, H. C. Beck, K. Becker, M. Becker, C. Becot, A. J. Beddall, A. Beddall, V. A. Bednyakov, M. Bedognetti, C. P. Bee, T. A. Beermann, M. Begalli, M. Begel, J. K. Behr, A. S. Bell, G. Bella, L. Bellagamba, A. Bellerive, M. Bellomo, K. Belotskiy, O. Beltramello, N. L. Belyaev, O. Benary, D. Benchekroun, M. Bender, K. Bendtz, N. Benekos, Y. Benhammou, E. Benhar Noccioli, J. Benitez, D. P. Benjamin, M. Benoit, J. R. Bensinger, S. Bentvelsen, L. Beresford, M. Beretta, D. Berge, E. Bergeaas Kuutmann, N. Berger, J. Beringer, S. Berlendis, N. R. Bernard, G. Bernardi, C. Bernius, F. U. Bernlochner, T. Berry, P. Berta, C. Bertella, G. Bertoli, F. Bertolucci, I. A. Bertram, C. Bertsche, D. Bertsche, G. J. Besjes, O. Bessidskaia Bylund, M. Bessner, N. Besson, A. Bethani, S. Bethke, A. J. Bevan, J. Beyer, R. M. Bianchi, O. Biebel, D. Biedermann, R. Bielski, K. Bierwagen, N. V. Biesuz, M. Biglietti, T. R. V. Billoud, H. Bilokon, M. Bindi, A. Bingul, C. Bini, S. Biondi, T. Bisanz, C. Bittrich, D. M. Bjergaard, J. E. Black, K. M. Black, R. E. Blair, T. Blazek, I. Bloch, C. Blocker, A. Blue, W. Blum, U. Blumenschein, S. Blunier, G. J. Bobbink, V. S. Bobrovnikov, S. S. Bocchetta, A. Bocci, C. Bock, M. Boehler, D. Boerner, D. Bogavac, A. G. Bogdanchikov, C. Bohm, V. Boisvert, P. Bokan, T. Bold, A. S. Boldyrev, A. E. Bolz, M. Bomben, M. Bona, M. Boonekamp, A. Borisov, G. Borissov, J. Bortfeldt, D. Bortoletto, V. Bortolotto, D. Boscherini, M. Bosman, J. D. Bossio Sola, J. Boudreau, J. Bouffard, E. V. Bouhova-Thacker, D. Boumediene, C. Bourdarios, S. K. Boutle, A. Boveia, J. Boyd, I. R. Boyko, A. J. Bozson, J. Bracinik, A. Brandt, G. Brandt, O. Brandt, U. Bratzler, B. Brau, J. E. Brau, W. D. Breaden Madden, K. Brendlinger, A. J. Brennan, L. Brenner, R. Brenner, S. Bressler, D. L. Briglin, T. M. Bristow, D. Britton, D. Britzger, F. M. Brochu, I. Brock, R. Brock, G. Brooijmans, T. Brooks, W. K. Brooks, J. Brosamer, E. Brost, J. H Broughton, P. A. Bruckman de Renstrom, D. Bruncko, A. Bruni, G. Bruni, L. S. Bruni, S. Bruno, BH Brunt, M. Bruschi, N. Bruscino, P. Bryant, L. Bryngemark, T. Buanes, Q. Buat, P. Buchholz, A. G. Buckley, I. A. Budagov, F. Buehrer, M. K. Bugge, O. Bulekov, D. Bullock, T. J. Burch, S. Burdin, C. D. Burgard, A. M. Burger, B. Burghgrave, K. Burka, S. Burke, I. Burmeister, J. T. P. Burr, E. Busato, D. Büscher, V. Büscher, P. Bussey, J. M. Butler, C. M. Buttar, J. M. Butterworth, P. Butti, W. Buttinger, A. Buzatu, A. R. Buzykaev, S. Cabrera Urbán, D. Caforio, V. M. Cairo, O. Cakir, N. Calace, P. Calafiura, A. Calandri, G. Calderini, P. Calfayan, G. Callea, L. P. Caloba, S. Calvente Lopez, D. Calvet, S. Calvet, T. P. Calvet, R. Camacho Toro, S. Camarda, P. Camarri, D. Cameron, R. Caminal Armadans, C. Camincher, S. Campana, M. Campanelli, A. Camplani, A. Campoverde, V. Canale, M. Cano Bret, J. Cantero, T. Cao, M. D. M. Capeans Garrido, I. Caprini, M. Caprini, M. Capua, R. M. Carbone, R. Cardarelli, F. Cardillo, I. Carli, T. Carli, G. Carlino, B. T. Carlson, L. Carminati, R. M. D. Carney, S. Caron, E. Carquin, S. Carrá, G. D. Carrillo-Montoya, D. Casadei, M. P. Casado, M. Casolino, D. W. Casper, R. Castelijn, V. Castillo Gimenez, N. F. Castro, A. Catinaccio, J. R. Catmore, A. Cattai, J. Caudron, V. Cavaliere, E. Cavallaro, D. Cavalli, M. Cavalli-Sforza, V. Cavasinni, E. Celebi, F. Ceradini, L. Cerda Alberich, A. S. Cerqueira, A. Cerri, L. Cerrito, F. Cerutti, A. Cervelli, S. A. Cetin, A. Chafaq, D. Chakraborty, S. K. Chan, W. S. Chan, Y. L. Chan, P. Chang, J. D. Chapman, D. G. Charlton, C. C. Chau, C. A. Chavez Barajas, S. Che, S. Cheatham, A. Chegwidden, S. Chekanov, S. V. Chekulaev, G. A. Chelkov, M. A. Chelstowska, C. Chen, C. Chen, H. Chen, J. Chen, S. Chen, S. Chen, X. Chen, Y. Chen, H. C. Cheng, H. J. Cheng, A. Cheplakov, E. Cheremushkina, R. Cherkaoui El Moursli, E. Cheu, K. Cheung, L. Chevalier, V. Chiarella, G. Chiarelli, G. Chiodini, A. S. Chisholm, A. Chitan, Y. H. Chiu, M. V. Chizhov, K. Choi, A. R. Chomont, S. Chouridou, Y. S. Chow, V. Christodoulou, M. C. Chu, J. Chudoba, A. J. Chuinard, J. J. Chwastowski, L. Chytka, A. K. Ciftci, D. Cinca, V. Cindro, I. A. Cioara, C. Ciocca, A. Ciocio, F. Cirotto, Z. H. Citron, M. Citterio, M. Ciubancan, A. Clark, B. L. Clark, M. R. Clark, P. J. Clark, R. N. Clarke, C. Clement, Y. Coadou, M. Cobal, A. Coccaro, J. Cochran, L. Colasurdo, B. Cole, A. P. Colijn, J. Collot, T. Colombo, P. Conde Muiño, E. Coniavitis, S. H. Connell, I. A. Connelly, S. Constantinescu, G. Conti, F. Conventi, M. Cooke, A. M. Cooper-Sarkar, F. Cormier, K. J. R. Cormier, M. Corradi, F. Corriveau, A. Cortes-Gonzalez, G. Cortiana, G. Costa, M. J. Costa, D. Costanzo, G. Cottin, G. Cowan, B. E. Cox, K. Cranmer, S. J. Crawley, R. A. Creager, G. Cree, S. Crépé-Renaudin, F. Crescioli, W. A. Cribbs, M. Cristinziani, V. Croft, G. Crosetti, A. Cueto, T. Cuhadar Donszelmann, A. R. Cukierman, J. Cummings, M. Curatolo, J. Cúth, S. Czekierda, P. Czodrowski, G. D’amen, S. D’Auria, L. D’eramo, M. D’Onofrio, M. J. Da Cunha Sargedas De Sousa, C. Da Via, W. Dabrowski, T. Dado, T. Dai, O. Dale, F. Dallaire, C. Dallapiccola, M. Dam, J. R. Dandoy, M. F. Daneri, N. P. Dang, A. C. Daniells, N. S. Dann, M. Danninger, M. Dano Hoffmann, V. Dao, G. Darbo, S. Darmora, J. Dassoulas, A. Dattagupta, T. Daubney, W. Davey, C. David, T. Davidek, D. R. Davis, P. Davison, E. Dawe, I. Dawson, K. De, R. de Asmundis, A. De Benedetti, S. De Castro, S. De Cecco, N. De Groot, P. de Jong, H. De la Torre, F. De Lorenzi, A. De Maria, D. De Pedis, A. De Salvo, U. De Sanctis, A. De Santo, K. De Vasconcelos Corga, J. B. De Vivie De Regie, R. Debbe, C. Debenedetti, D. V. Dedovich, N. Dehghanian, I. Deigaard, M. Del Gaudio, J. Del Peso, D. Delgove, F. Deliot, C. M. Delitzsch, A. Dell’Acqua, L. Dell’Asta, M. Dell’Orso, M. Della Pietra, D. della Volpe, M. Delmastro, C. Delporte, P. A. Delsart, D. A. DeMarco, S. Demers, M. Demichev, A. Demilly, S. P. Denisov, D. Denysiuk, D. Derendarz, J. E. Derkaoui, F. Derue, P. Dervan, K. Desch, C. Deterre, K. Dette, M. R. Devesa, P. O. Deviveiros, A. Dewhurst, S. Dhaliwal, F. A. Di Bello, A. Di Ciaccio, L. Di Ciaccio, W. K. Di Clemente, C. Di Donato, A. Di Girolamo, B. Di Girolamo, B. Di Micco, R. Di Nardo, K. F. Di Petrillo, A. Di Simone, R. Di Sipio, D. Di Valentino, C. Diaconu, M. Diamond, F. A. Dias, M. A. Diaz, E. B. Diehl, J. Dietrich, S. Díez Cornell, A. Dimitrievska, J. Dingfelder, P. Dita, S. Dita, F. Dittus, F. Djama, T. Djobava, J. I. Djuvsland, M. A. B. do Vale, D. Dobos, M. Dobre, C. Doglioni, J. Dolejsi, Z. Dolezal, M. Donadelli, S. Donati, P. Dondero, J. Donini, J. Dopke, A. Doria, M. T. Dova, A. T. Doyle, E. Drechsler, M. Dris, Y. Du, J. Duarte-Campderros, A. Dubreuil, E. Duchovni, G. Duckeck, A. Ducourthial, O. A. Ducu, D. Duda, A. Dudarev, A. Chr. Dudder, E. M. Duffield, L. Duflot, M. Dührssen, C. Dulsen, M. Dumancic, A. E. Dumitriu, A. K. Duncan, M. Dunford, H. Duran Yildiz, M. Düren, A. Durglishvili, D. Duschinger, B. Dutta, D. Duvnjak, M. Dyndal, B. S. Dziedzic, C. Eckardt, K. M. Ecker, R. C. Edgar, T. Eifert, G. Eigen, K. Einsweiler, T. Ekelof, M. El Kacimi, R. El Kosseifi, V. Ellajosyula, M. Ellert, S. Elles, F. Ellinghaus, A. A. Elliot, N. Ellis, J. Elmsheuser, M. Elsing, D. Emeliyanov, Y. Enari, O. C. Endner, J. S. Ennis, J. Erdmann, A. Ereditato, M. Ernst, S. Errede, M. Escalier, C. Escobar, B. Esposito, O. Estrada Pastor, A. I. Etienvre, E. Etzion, H. Evans, A. Ezhilov, M. Ezzi, F. Fabbri, L. Fabbri, V. Fabiani, G. Facini, R. M. Fakhrutdinov, S. Falciano, R. J. Falla, J. Faltova, Y. Fang, M. Fanti, A. Farbin, A. Farilla, C. Farina, E. M. Farina, T. Farooque, S. Farrell, S. M. Farrington, P. Farthouat, F. Fassi, P. Fassnacht, D. Fassouliotis, M. Faucci Giannelli, A. Favareto, W. J. Fawcett, L. Fayard, O. L. Fedin, W. Fedorko, S. Feigl, L. Feligioni, C. Feng, E. J. Feng, H. Feng, M. J. Fenton, A. B. Fenyuk, L. Feremenga, P. Fernandez Martinez, S. Fernandez Perez, J. Ferrando, A. Ferrari, P. Ferrari, R. Ferrari, D. E. Ferreira de Lima, A. Ferrer, D. Ferrere, C. Ferretti, F. Fiedler, A. Filipčič, M. Filipuzzi, F. Filthaut, M. Fincke-Keeler, K. D. Finelli, M. C. N. Fiolhais, L. Fiorini, A. Fischer, C. Fischer, J. Fischer, W. C. Fisher, N. Flaschel, I. Fleck, P. Fleischmann, R. R. M. Fletcher, T. Flick, B. M. Flierl, L. R. Flores Castillo, M. J. Flowerdew, G. T. Forcolin, A. Formica, F. A. Förster, A. Forti, A. G. Foster, D. Fournier, H. Fox, S. Fracchia, P. Francavilla, M. Franchini, S. Franchino, D. Francis, L. Franconi, M. Franklin, M. Frate, M. Fraternali, D. Freeborn, S. M. Fressard-Batraneanu, B. Freund, D. Froidevaux, J. A. Frost, C. Fukunaga, T. Fusayasu, J. Fuster, C. Gabaldon, O. Gabizon, A. Gabrielli, A. Gabrielli, G. P. Gach, S. Gadatsch, S. Gadomski, G. Gagliardi, L. G. Gagnon, C. Galea, B. Galhardo, E. J. Gallas, B. J. Gallop, P. Gallus, G. Galster, K. K. Gan, S. Ganguly, Y. Gao, Y. S. Gao, F. M. Garay Walls, C. García, J. E. García Navarro, J. A. García Pascual, M. Garcia-Sciveres, R. W. Gardner, N. Garelli, V. Garonne, A. Gascon Bravo, K. Gasnikova, C. Gatti, A. Gaudiello, G. Gaudio, I. L. Gavrilenko, C. Gay, G. Gaycken, E. N. Gazis, C. N. P. Gee, J. Geisen, M. Geisen, M. P. Geisler, K. Gellerstedt, C. Gemme, M. H. Genest, C. Geng, S. Gentile, C. Gentsos, S. George, D. Gerbaudo, A. Gershon, G. Geßner, S. Ghasemi, M. Ghneimat, B. Giacobbe, S. Giagu, N. Giangiacomi, P. Giannetti, S. M. Gibson, M. Gignac, M. Gilchriese, D. Gillberg, G. Gilles, D. M. Gingrich, M. P. Giordani, F. M. Giorgi, P. F. Giraud, P. Giromini, G. Giugliarelli, D. Giugni, F. Giuli, C. Giuliani, M. Giulini, B. K. Gjelsten, S. Gkaitatzis, I. Gkialas, E. L. Gkougkousis, P. Gkountoumis, L. K. Gladilin, C. Glasman, J. Glatzer, P. C. F. Glaysher, A. Glazov, M. Goblirsch-Kolb, J. Godlewski, S. Goldfarb, T. Golling, D. Golubkov, A. Gomes, R. Gonçalo, R. Goncalves Gama, J. Goncalves Pinto Firmino Da Costa, G. Gonella, L. Gonella, A. Gongadze, S. González de la Hoz, S. Gonzalez-Sevilla, L. Goossens, P. A. Gorbounov, H. A. Gordon, I. Gorelov, B. Gorini, E. Gorini, A. Gorišek, A. T. Goshaw, C. Gössling, M. I. Gostkin, C. A. Gottardo, C. R. Goudet, D. Goujdami, A. G. Goussiou, N. Govender, E. Gozani, L. Graber, I. Grabowska-Bold, P. O. J. Gradin, J. Gramling, E. Gramstad, S. Grancagnolo, V. Gratchev, P. M. Gravila, C. Gray, H. M. Gray, Z. D. Greenwood, C. Grefe, K. Gregersen, I. M. Gregor, P. Grenier, K. Grevtsov, J. Griffiths, A. A. Grillo, K. Grimm, S. Grinstein, Ph. Gris, J.-F. Grivaz, S. Groh, E. Gross, J. Grosse-Knetter, G. C. Grossi, Z. J. Grout, A. Grummer, L. Guan, W. Guan, J. Guenther, F. Guescini, D. Guest, O. Gueta, B. Gui, E. Guido, T. Guillemin, S. Guindon, U. Gul, C. Gumpert, J. Guo, W. Guo, Y. Guo, R. Gupta, S. Gupta, G. Gustavino, B. J. Gutelman, P. Gutierrez, N. G. Gutierrez Ortiz, C. Gutschow, C. Guyot, M. P. Guzik, C. Gwenlan, C. B. Gwilliam, A. Haas, C. Haber, H. K. Hadavand, N. Haddad, A. Hadef, S. Hageböck, M. Hagihara, H. Hakobyan, M. Haleem, J. Haley, G. Halladjian, G. D. Hallewell, K. Hamacher, P. Hamal, K. Hamano, A. Hamilton, G. N. Hamity, P. G. Hamnett, L. Han, S. Han, K. Hanagaki, K. Hanawa, M. Hance, B. Haney, P. Hanke, J. B. Hansen, J. D. Hansen, M. C. Hansen, P. H. Hansen, K. Hara, A. S. Hard, T. Harenberg, F. Hariri, S. Harkusha, P. F. Harrison, N. M. Hartmann, Y. Hasegawa, A. Hasib, S. Hassani, S. Haug, R. Hauser, L. Hauswald, L. B. Havener, M. Havranek, C. M. Hawkes, R. J. Hawkings, D. Hayakawa, D. Hayden, C. P. Hays, J. M. Hays, H. S. Hayward, S. J. Haywood, S. J. Head, T. Heck, V. Hedberg, L. Heelan, S. Heer, K. K. Heidegger, S. Heim, T. Heim, B. Heinemann, J. J. Heinrich, L. Heinrich, C. Heinz, J. Hejbal, L. Helary, A. Held, S. Hellman, C. Helsens, R. C. W. Henderson, Y. Heng, S. Henkelmann, A. M. Henriques Correia, S. Henrot-Versille, G. H. Herbert, H. Herde, V. Herget, Y. Hernández Jiménez, H. Herr, G. Herten, R. Hertenberger, L. Hervas, T. C. Herwig, G. G. Hesketh, N. P. Hessey, J. W. Hetherly, S. Higashino, E. Higón-Rodriguez, K. Hildebrand, E. Hill, J. C. Hill, K. H. Hiller, S. J. Hillier, M. Hils, I. Hinchliffe, M. Hirose, D. Hirschbuehl, B. Hiti, O. Hladik, X. Hoad, J. Hobbs, N. Hod, M. C. Hodgkinson, P. Hodgson, A. Hoecker, M. R. Hoeferkamp, F. Hoenig, D. Hohn, T. R. Holmes, M. Homann, S. Honda, T. Honda, T. M. Hong, B. H. Hooberman, W. H. Hopkins, Y. Horii, A. J. Horton, J-Y. Hostachy, A. Hostiuc, S. Hou, A. Hoummada, J. Howarth, J. Hoya, M. Hrabovsky, J. Hrdinka, I. Hristova, J. Hrivnac, T. Hryn’ova, A. Hrynevich, P. J. Hsu, S.-C. Hsu, Q. Hu, S. Hu, Y. Huang, Z. Hubacek, F. Hubaut, F. Huegging, T. B. Huffman, E. W. Hughes, G. Hughes, M. Huhtinen, P. Huo, N. Huseynov, J. Huston, J. Huth, G. Iacobucci, G. Iakovidis, I. Ibragimov, L. Iconomidou-Fayard, Z. Idrissi, P. Iengo, O. Igonkina, T. Iizawa, Y. Ikegami, M. Ikeno, Y. Ilchenko, D. Iliadis, N. Ilic, G. Introzzi, P. Ioannou, M. Iodice, K. Iordanidou, V. Ippolito, M. F. Isacson, N. Ishijima, M. Ishino, M. Ishitsuka, C. Issever, S. Istin, F. Ito, J. M. Iturbe Ponce, R. Iuppa, H. Iwasaki, J. M. Izen, V. Izzo, S. Jabbar, P. Jackson, R. M. Jacobs, V. Jain, K. B. Jakobi, K. Jakobs, S. Jakobsen, T. Jakoubek, D. O. Jamin, D. K. Jana, R. Jansky, J. Janssen, M. Janus, P. A. Janus, G. Jarlskog, N. Javadov, T. Javůrek, M. Javurkova, F. Jeanneau, L. Jeanty, J. Jejelava, A. Jelinskas, P. Jenni, C. Jeske, S. Jézéquel, H. Ji, J. Jia, H. Jiang, Y. Jiang, Z. Jiang, S. Jiggins, J. Jimenez Pena, S. Jin, A. Jinaru, O. Jinnouchi, H. Jivan, P. Johansson, K. A. Johns, C. A. Johnson, W. J. Johnson, K. Jon-And, R. W. L. Jones, S. D. Jones, S. Jones, T. J. Jones, J. Jongmanns, P. M. Jorge, J. Jovicevic, X. Ju, A. Juste Rozas, M. K. Köhler, A. Kaczmarska, M. Kado, H. Kagan, M. Kagan, S. J. Kahn, T. Kaji, E. Kajomovitz, C. W. Kalderon, A. Kaluza, S. Kama, A. Kamenshchikov, N. Kanaya, L. Kanjir, V. A. Kantserov, J. Kanzaki, B. Kaplan, L. S. Kaplan, D. Kar, K. Karakostas, N. Karastathis, M. J. Kareem, E. Karentzos, S. N. Karpov, Z. M. Karpova, K. Karthik, V. Kartvelishvili, A. N. Karyukhin, K. Kasahara, L. Kashif, R. D. Kass, A. Kastanas, Y. Kataoka, C. Kato, A. Katre, J. Katzy, K. Kawade, K. Kawagoe, T. Kawamoto, G. Kawamura, E. F. Kay, V. F. Kazanin, R. Keeler, R. Kehoe, J. S. Keller, E. Kellermann, J. J. Kempster, J Kendrick, H. Keoshkerian, O. Kepka, B. P. Kerševan, S. Kersten, R. A. Keyes, M. Khader, F. Khalil-zada, A. Khanov, A. G. Kharlamov, T. Kharlamova, A. Khodinov, T. J. Khoo, V. Khovanskiy, E. Khramov, J. Khubua, S. Kido, C. R. Kilby, H. Y. Kim, S. H. Kim, Y. K. Kim, N. Kimura, O. M. Kind, B. T. King, D. Kirchmeier, J. Kirk, A. E. Kiryunin, T. Kishimoto, D. Kisielewska, V. Kitali, O. Kivernyk, E. Kladiva, T. Klapdor-Kleingrothaus, M. H. Klein, M. Klein, U. Klein, K. Kleinknecht, P. Klimek, A. Klimentov, R. Klingenberg, T. Klingl, T. Klioutchnikova, E.-E. Kluge, P. Kluit, S. Kluth, E. Kneringer, E. B. F. G. Knoops, A. Knue, A. Kobayashi, D. Kobayashi, T. Kobayashi, M. Kobel, M. Kocian, P. Kodys, T. Koffas, E. Koffeman, N. M. Köhler, T. Koi, M. Kolb, I. Koletsou, A. A. Komar, T. Kondo, N. Kondrashova, K. Köneke, A. C. König, T. Kono, R. Konoplich, N. Konstantinidis, R. Kopeliansky, S. Koperny, A. K. Kopp, K. Korcyl, K. Kordas, A. Korn, A. A. Korol, I. Korolkov, E. V. Korolkova, O. Kortner, S. Kortner, T. Kosek, V. V. Kostyukhin, A. Kotwal, A. Koulouris, A. Kourkoumeli-Charalampidi, C. Kourkoumelis, E. Kourlitis, V. Kouskoura, A. B. Kowalewska, R. Kowalewski, T. Z. Kowalski, C. Kozakai, W. Kozanecki, A. S. Kozhin, V. A. Kramarenko, G. Kramberger, D. Krasnopevtsev, M. W. Krasny, A. Krasznahorkay, D. Krauss, J. A. Kremer, J. Kretzschmar, K. Kreutzfeldt, P. Krieger, K. Krizka, K. Kroeninger, H. Kroha, J. Kroll, J. Kroll, J. Kroseberg, J. Krstic, U. Kruchonak, H. Krüger, N. Krumnack, M. C. Kruse, T. Kubota, H. Kucuk, S. Kuday, J. T. Kuechler, S. Kuehn, A. Kugel, F. Kuger, T. Kuhl, V. Kukhtin, R. Kukla, Y. Kulchitsky, S. Kuleshov, Y. P. Kulinich, M. Kuna, T. Kunigo, A. Kupco, T. Kupfer, O. Kuprash, H. Kurashige, L. L. Kurchaninov, Y. A. Kurochkin, M. G. Kurth, V. Kus, E. S. Kuwertz, M. Kuze, J. Kvita, T. Kwan, D. Kyriazopoulos, A. La Rosa, J. L. La Rosa Navarro, L. La Rotonda, F. La Ruffa, C. Lacasta, F. Lacava, J. Lacey, D. P. J. Lack, H. Lacker, D. Lacour, E. Ladygin, R. Lafaye, B. Laforge, T. Lagouri, S. Lai, S. Lammers, W. Lampl, E. Lançon, U. Landgraf, M. P. J. Landon, M. C. Lanfermann, V. S. Lang, J. C. Lange, R. J. Langenberg, A. J. Lankford, F. Lanni, K. Lantzsch, A. Lanza, A. Lapertosa, S. Laplace, J. F. Laporte, T. Lari, F. Lasagni Manghi, M. Lassnig, T. S. Lau, P. Laurelli, W. Lavrijsen, A. T. Law, P. Laycock, T. Lazovich, M. Lazzaroni, B. Le, O. Le Dortz, E. Le Guirriec, E. P. Le Quilleuc, M. LeBlanc, T. LeCompte, F. Ledroit-Guillon, C. A. Lee, G. R. Lee, S. C. Lee, L. Lee, B. Lefebvre, G. Lefebvre, M. Lefebvre, F. Legger, C. Leggett, G. Lehmann Miotto, X. Lei, W. A. Leight, M. A. L. Leite, R. Leitner, D. Lellouch, B. Lemmer, K. J. C. Leney, T. Lenz, B. Lenzi, R. Leone, S. Leone, C. Leonidopoulos, G. Lerner, C. Leroy, A. A. J. Lesage, C. G. Lester, M. Levchenko, J. Levêque, D. Levin, L. J. Levinson, M. Levy, D. Lewis, B. Li, Changqiao Li, H. Li, L. Li, Q. Li, Q. Li, S. Li, X. Li, Y. Li, Z. Liang, B. Liberti, A. Liblong, K. Lie, J. Liebal, W. Liebig, A. Limosani, S. C. Lin, T. H. Lin, R. A. Linck, B. E. Lindquist, A. E. Lionti, E. Lipeles, A. Lipniacka, M. Lisovyi, T. M. Liss, A. Lister, A. M. Litke, B. Liu, H. Liu, H. Liu, J. K. K. Liu, J. Liu, J. B. Liu, K. Liu, L. Liu, M. Liu, Y. L. Liu, Y. Liu, M. Livan, A. Lleres, J. Llorente Merino, S. L. Lloyd, C. Y. Lo, F. Lo Sterzo, E. M. Lobodzinska, P. Loch, F. K. Loebinger, A. Loesle, K. M. Loew, A. Loginov, T. Lohse, K. Lohwasser, M. Lokajicek, B. A. Long, J. D. Long, R. E. Long, L. Longo, K. A. Looper, J. A. Lopez, D. Lopez Mateos, I. Lopez Paz, A. Lopez Solis, J. Lorenz, N. Lorenzo Martinez, M. Losada, P. J. Lösel, X. Lou, A. Lounis, J. Love, P. A. Love, H. Lu, N. Lu, Y. J. Lu, H. J. Lubatti, C. Luci, A. Lucotte, C. Luedtke, F. Luehring, W. Lukas, L. Luminari, O. Lundberg, B. Lund-Jensen, M. S. Lutz, P. M. Luzi, D. Lynn, R. Lysak, E. Lytken, F. Lyu, V. Lyubushkin, H. Ma, L. L. Ma, Y. Ma, G. Maccarrone, A. Macchiolo, C. M. Macdonald, B. Maček, J. Machado Miguens, D. Madaffari, R. Madar, W. F. Mader, A. Madsen, J. Maeda, S. Maeland, T. Maeno, A. S. Maevskiy, V. Magerl, J. Mahlstedt, C. Maiani, C. Maidantchik, A. A. Maier, T. Maier, A. Maio, O. Majersky, S. Majewski, Y. Makida, N. Makovec, B. Malaescu, Pa. Malecki, V. P. Maleev, F. Malek, U. Mallik, D. Malon, C. Malone, S. Maltezos, S. Malyukov, J. Mamuzic, G. Mancini, I. Mandić, J. Maneira, L. Manhaes de Andrade Filho, J. Manjarres Ramos, K. H. Mankinen, A. Mann, A. Manousos, B. Mansoulie, J. D. Mansour, R. Mantifel, M. Mantoani, S. Manzoni, L. Mapelli, G. Marceca, L. March, L. Marchese, G. Marchiori, M. Marcisovsky, C. A. Marin Tobon, M. Marjanovic, D. E. Marley, F. Marroquim, S. P. Marsden, Z. Marshall, M.U.F Martensson, S. Marti-Garcia, C. B. Martin, T. A. Martin, V. J. Martin, B. Martin dit Latour, M. Martinez, V. I. Martinez Outschoorn, S. Martin-Haugh, V. S. Martoiu, A. C. Martyniuk, A. Marzin, L. Masetti, T. Mashimo, R. Mashinistov, J. Masik, A. L. Maslennikov, L. Massa, P. Mastrandrea, A. Mastroberardino, T. Masubuchi, P. Mättig, J. Maurer, S. J. Maxfield, D. A. Maximov, R. Mazini, I. Maznas, S. M. Mazza, N. C. Mc Fadden, G. Mc Goldrick, S. P. Mc Kee, A. McCarn, R. L. McCarthy, T. G. McCarthy, L. I. McClymont, E. F. McDonald, J. A. Mcfayden, G. Mchedlidze, S. J. McMahon, P. C. McNamara, C. J. McNicol, R. A. McPherson, S. Meehan, T. J. Megy, S. Mehlhase, A. Mehta, T. Meideck, K. Meier, B. Meirose, D. Melini, B. R. Mellado Garcia, J. D. Mellenthin, M. Melo, F. Meloni, A. Melzer, S. B. Menary, L. Meng, X. T. Meng, A. Mengarelli, S. Menke, E. Meoni, S. Mergelmeyer, C. Merlassino, P. Mermod, L. Merola, C. Meroni, F. S. Merritt, A. Messina, J. Metcalfe, A. S. Mete, C. Meyer, J-P. Meyer, J. Meyer, H. Meyer Zu Theenhausen, F. Miano, R. P. Middleton, S. Miglioranzi, L. Mijović, G. Mikenberg, M. Mikestikova, M. Mikuž, M. Milesi, A. Milic, D. A. Millar, D. W. Miller, C. Mills, A. Milov, D. A. Milstead, A. A. Minaenko, Y. Minami, I. A. Minashvili, A. I. Mincer, B. Mindur, M. Mineev, Y. Minegishi, Y. Ming, L. M. Mir, K. P. Mistry, T. Mitani, J. Mitrevski, V. A. Mitsou, A. Miucci, P. S. Miyagawa, A. Mizukami, J. U. Mjörnmark, T. Mkrtchyan, M. Mlynarikova, T. Moa, K. Mochizuki, P. Mogg, S. Mohapatra, S. Molander, R. Moles-Valls, M. C. Mondragon, K. Mönig, J. Monk, E. Monnier, A. Montalbano, J. Montejo Berlingen, F. Monticelli, S. Monzani, R. W. Moore, N. Morange, D. Moreno, M. Moreno Llácer, P. Morettini, S. Morgenstern, D. Mori, T. Mori, M. Morii, M. Morinaga, V. Morisbak, A. K. Morley, G. Mornacchi, J. D. Morris, L. Morvaj, P. Moschovakos, M. Mosidze, H. J. Moss, J. Moss, K. Motohashi, R. Mount, E. Mountricha, E. J. W. Moyse, S. Muanza, F. Mueller, J. Mueller, R. S. P. Mueller, D. Muenstermann, P. Mullen, G. A. Mullier, F. J. Munoz Sanchez, W. J. Murray, H. Musheghyan, M. Muškinja, A. G. Myagkov, M. Myska, B. P. Nachman, O. Nackenhorst, K. Nagai, R. Nagai, K. Nagano, Y. Nagasaka, K. Nagata, M. Nagel, E. Nagy, A. M. Nairz, Y. Nakahama, K. Nakamura, T. Nakamura, I. Nakano, R. F. Naranjo Garcia, R. Narayan, D. I. Narrias Villar, I. Naryshkin, T. Naumann, G. Navarro, R. Nayyar, H. A. Neal, P. Yu. Nechaeva, T. J. Neep, A. Negri, M. Negrini, S. Nektarijevic, C. Nellist, A. Nelson, M. E. Nelson, S. Nemecek, P. Nemethy, M. Nessi, M. S. Neubauer, M. Neumann, P. R. Newman, T. Y. Ng, T. Nguyen Manh, R. B. Nickerson, R. Nicolaidou, J. Nielsen, V. Nikolaenko, I. Nikolic-Audit, K. Nikolopoulos, J. K. Nilsen, P. Nilsson, Y. Ninomiya, A. Nisati, N. Nishu, R. Nisius, I. Nitsche, T. Nitta, T. Nobe, Y. Noguchi, M. Nomachi, I. Nomidis, M. A. Nomura, T. Nooney, M. Nordberg, N. Norjoharuddeen, O. Novgorodova, M. Nozaki, L. Nozka, K. Ntekas, E. Nurse, F. Nuti, K. O’connor, D. C. O’Neil, A. A. O’Rourke, V. O’Shea, F. G. Oakham, H. Oberlack, T. Obermann, J. Ocariz, A. Ochi, I. Ochoa, J. P. Ochoa-Ricoux, S. Oda, S. Odaka, A. Oh, S. H. Oh, C. C. Ohm, H. Ohman, H. Oide, H. Okawa, Y. Okumura, T. Okuyama, A. Olariu, L. F. Oleiro Seabra, S. A. Olivares Pino, D. Oliveira Damazio, A. Olszewski, J. Olszowska, A. Onofre, K. Onogi, P. U. E. Onyisi, H. Oppen, M. J. Oreglia, Y. Oren, D. Orestano, N. Orlando, R. S. Orr, B. Osculati, R. Ospanov, G. Otero y Garzon, H. Otono, M. Ouchrif, F. Ould-Saada, A. Ouraou, K. P. Oussoren, Q. Ouyang, M. Owen, R. E. Owen, V. E. Ozcan, N. Ozturk, K. Pachal, A. Pacheco Pages, L. Pacheco Rodriguez, C. Padilla Aranda, S. Pagan Griso, M. Paganini, F. Paige, G. Palacino, S. Palazzo, S. Palestini, M. Palka, D. Pallin, E. St. Panagiotopoulou, I. Panagoulias, C. E. Pandini, J. G. Panduro Vazquez, P. Pani, S. Panitkin, D. Pantea, L. Paolozzi, Th. D. Papadopoulou, K. Papageorgiou, A. Paramonov, D. Paredes Hernandez, A. J. Parker, M. A. Parker, K. A. Parker, F. Parodi, J. A. Parsons, U. Parzefall, V. R. Pascuzzi, J. M. Pasner, E. Pasqualucci, S. Passaggio, Fr. Pastore, S. Pataraia, J. R. Pater, T. Pauly, B. Pearson, S. Pedraza Lopez, R. Pedro, S. V. Peleganchuk, O. Penc, C. Peng, H. Peng, J. Penwell, B. S. Peralva, M. M. Perego, D. V. Perepelitsa, F. Peri, L. Perini, H. Pernegger, S. Perrella, R. Peschke, V. D. Peshekhonov, K. Peters, R. F. Y. Peters, B. A. Petersen, T. C. Petersen, E. Petit, A. Petridis, C. Petridou, P. Petroff, E. Petrolo, M. Petrov, F. Petrucci, N. E. Pettersson, A. Peyaud, R. Pezoa, F. H. Phillips, P. W. Phillips, G. Piacquadio, E. Pianori, A. Picazio, E. Piccaro, M. A. Pickering, R. Piegaia, J. E. Pilcher, A. D. Pilkington, A. W. J. Pin, M. Pinamonti, J. L. Pinfold, H. Pirumov, M. Pitt, L. Plazak, M.-A. Pleier, V. Pleskot, E. Plotnikova, D. Pluth, P. Podberezko, R. Poettgen, R. Poggi, L. Poggioli, I. Pogrebnyak, D. Pohl, G. Polesello, A. Poley, A. Policicchio, R. Polifka, A. Polini, C. S. Pollard, V. Polychronakos, K. Pommès, D. Ponomarenko, L. Pontecorvo, G. A. Popeneciu, D. M. Portillo Quintero, S. Pospisil, K. Potamianos, I. N. Potrap, C. J. Potter, H. Potti, T. Poulsen, J. Poveda, M. E. Pozo Astigarraga, P. Pralavorio, A. Pranko, S. Prell, D. Price, M. Primavera, S. Prince, N. Proklova, K. Prokofiev, F. Prokoshin, S. Protopopescu, J. Proudfoot, M. Przybycien, A. Puri, P. Puzo, J. Qian, G. Qin, Y. Qin, A. Quadt, M. Queitsch-Maitland, D. Quilty, S. Raddum, V. Radeka, V. Radescu, S. K. Radhakrishnan, P. Radloff, P. Rados, F. Ragusa, G. Rahal, J. A. Raine, S. Rajagopalan, C. Rangel-Smith, T. Rashid, S. Raspopov, M. G. Ratti, D. M. Rauch, F. Rauscher, S. Rave, I. Ravinovich, J. H. Rawling, M. Raymond, A. L. Read, N. P. Readioff, M. Reale, D. M. Rebuzzi, A. Redelbach, G. Redlinger, R. Reece, R. G. Reed, K. Reeves, L. Rehnisch, J. Reichert, A. Reiss, C. Rembser, H. Ren, M. Rescigno, S. Resconi, E. D. Resseguie, S. Rettie, E. Reynolds, O. L. Rezanova, P. Reznicek, R. Rezvani, R. Richter, S. Richter, E. Richter-Was, O. Ricken, M. Ridel, P. Rieck, C. J. Riegel, J. Rieger, O. Rifki, M. Rijssenbeek, A. Rimoldi, M. Rimoldi, L. Rinaldi, G. Ripellino, B. Ristić, E. Ritsch, I. Riu, F. Rizatdinova, E. Rizvi, C. Rizzi, R. T. Roberts, S. H. Robertson, A. Robichaud-Veronneau, D. Robinson, J. E. M. Robinson, A. Robson, E. Rocco, C. Roda, Y. Rodina, S. Rodriguez Bosca, A. Rodriguez Perez, D. Rodriguez Rodriguez, S. Roe, C. S. Rogan, O. Røhne, J. Roloff, A. Romaniouk, M. Romano, S. M. Romano Saez, E. Romero Adam, N. Rompotis, M. Ronzani, L. Roos, S. Rosati, K. Rosbach, P. Rose, N.-A. Rosien, E. Rossi, L. P. Rossi, J. H. N. Rosten, R. Rosten, M. Rotaru, J. Rothberg, D. Rousseau, A. Rozanov, Y. Rozen, X. Ruan, F. Rubbo, F. Rühr, A. Ruiz-Martinez, Z. Rurikova, N. A. Rusakovich, H. L. Russell, J. P. Rutherfoord, N. Ruthmann, Y. F. Ryabov, M. Rybar, G. Rybkin, S. Ryu, A. Ryzhov, G. F. Rzehorz, A. F. Saavedra, G. Sabato, S. Sacerdoti, H. F-W. Sadrozinski, R. Sadykov, F. Safai Tehrani, P. Saha, M. Sahinsoy, M. Saimpert, M. Saito, T. Saito, H. Sakamoto, Y. Sakurai, G. Salamanna, J. E. Salazar Loyola, D. Salek, P. H. Sales De Bruin, D. Salihagic, A. Salnikov, J. Salt, D. Salvatore, F. Salvatore, A. Salvucci, A. Salzburger, D. Sammel, D. Sampsonidis, D. Sampsonidou, J. Sánchez, V. Sanchez Martinez, A. Sanchez Pineda, H. Sandaker, R. L. Sandbach, C. O. Sander, M. Sandhoff, C. Sandoval, D. P. C. Sankey, M. Sannino, Y. Sano, A. Sansoni, C. Santoni, H. Santos, I. Santoyo Castillo, A. Sapronov, J. G. Saraiva, B. Sarrazin, O. Sasaki, K. Sato, E. Sauvan, G. Savage, P. Savard, N. Savic, C. Sawyer, L. Sawyer, J. Saxon, C. Sbarra, A. Sbrizzi, T. Scanlon, D. A. Scannicchio, J. Schaarschmidt, P. Schacht, B. M. Schachtner, D. Schaefer, L. Schaefer, R. Schaefer, J. Schaeffer, S. Schaepe, S. Schaetzel, U. Schäfer, A. C. Schaffer, D. Schaile, R. D. Schamberger, V. A. Schegelsky, D. Scheirich, M. Schernau, C. Schiavi, S. Schier, L. K. Schildgen, C. Schillo, M. Schioppa, S. Schlenker, K. R. Schmidt-Sommerfeld, K. Schmieden, C. Schmitt, S. Schmitt, S. Schmitz, U. Schnoor, L. Schoeffel, A. Schoening, B. D. Schoenrock, E. Schopf, M. Schott, J. F. P. Schouwenberg, J. Schovancova, S. Schramm, N. Schuh, A. Schulte, M. J. Schultens, H.-C. Schultz-Coulon, H. Schulz, M. Schumacher, B. A. Schumm, Ph. Schune, A. Schwartzman, T. A. Schwarz, H. Schweiger, Ph. Schwemling, R. Schwienhorst, J. Schwindling, A. Sciandra, G. Sciolla, M. Scornajenghi, F. Scuri, F. Scutti, J. Searcy, P. Seema, S. C. Seidel, A. Seiden, J. M. Seixas, G. Sekhniaidze, K. Sekhon, S. J. Sekula, N. Semprini-Cesari, S. Senkin, C. Serfon, L. Serin, L. Serkin, M. Sessa, R. Seuster, H. Severini, T. Sfiligoj, F. Sforza, A. Sfyrla, E. Shabalina, N. W. Shaikh, L. Y. Shan, R. Shang, J. T. Shank, M. Shapiro, P. B. Shatalov, K. Shaw, S. M. Shaw, A. Shcherbakova, C. Y. Shehu, Y. Shen, N. Sherafati, P. Sherwood, L. Shi, S. Shimizu, C. O. Shimmin, M. Shimojima, I. P. J. Shipsey, S. Shirabe, M. Shiyakova, J. Shlomi, A. Shmeleva, D. Shoaleh Saadi, M. J. Shochet, S. Shojaii, D. R. Shope, S. Shrestha, E. Shulga, M. A. Shupe, P. Sicho, A. M. Sickles, P. E. Sidebo, E. Sideras Haddad, O. Sidiropoulou, A. Sidoti, F. Siegert, Dj. Sijacki, J. Silva, S. B. Silverstein, V. Simak, L. Simic, S. Simion, E. Simioni, B. Simmons, M. Simon, P. Sinervo, N. B. Sinev, M. Sioli, G. Siragusa, I. Siral, S. Yu. Sivoklokov, J. Sjölin, M. B. Skinner, P. Skubic, M. Slater, T. Slavicek, M. Slawinska, K. Sliwa, R. Slovak, V. Smakhtin, B. H. Smart, J. Smiesko, N. Smirnov, S. Yu. Smirnov, Y. Smirnov, L. N. Smirnova, O. Smirnova, J. W. Smith, M. N. K. Smith, R. W. Smith, M. Smizanska, K. Smolek, A. A. Snesarev, I. M. Snyder, S. Snyder, R. Sobie, F. Socher, A. Soffer, A. Søgaard, D. A. Soh, G. Sokhrannyi, C. A. Solans Sanchez, M. Solar, E. Yu. Soldatov, U. Soldevila, A. A. Solodkov, A. Soloshenko, O. V. Solovyanov, V. Solovyev, P. Sommer, H. Son, A. Sopczak, D. Sosa, C. L. Sotiropoulou, R. Soualah, A. M. Soukharev, D. South, B. C. Sowden, S. Spagnolo, M. Spalla, M. Spangenberg, F. Spanò, D. Sperlich, F. Spettel, T. M. Spieker, R. Spighi, G. Spigo, L. A. Spiller, M. Spousta, R. D. St. Denis, A. Stabile, R. Stamen, S. Stamm, E. Stanecka, R. W. Stanek, C. Stanescu, M. M. Stanitzki, B. S. Stapf, S. Stapnes, E. A. Starchenko, G. H. Stark, J. Stark, S. H Stark, P. Staroba, P. Starovoitov, S. Stärz, R. Staszewski, M. Stegler, P. Steinberg, B. Stelzer, H. J. Stelzer, O. Stelzer-Chilton, H. Stenzel, G. A. Stewart, M. C. Stockton, M. Stoebe, G. Stoicea, P. Stolte, S. Stonjek, A. R. Stradling, A. Straessner, M. E. Stramaglia, J. Strandberg, S. Strandberg, M. Strauss, P. Strizenec, R. Ströhmer, D. M. Strom, R. Stroynowski, A. Strubig, S. A. Stucci, B. Stugu, N. A. Styles, D. Su, J. Su, S. Suchek, Y. Sugaya, M. Suk, V. V. Sulin, DMS Sultan, S. Sultansoy, T. Sumida, S. Sun, X. Sun, K. Suruliz, C. J. E. Suster, M. R. Sutton, S. Suzuki, M. Svatos, M. Swiatlowski, S. P. Swift, I. Sykora, T. Sykora, D. Ta, K. Tackmann, J. Taenzer, A. Taffard, R. Tafirout, E. Tahirovic, N. Taiblum, H. Takai, R. Takashima, E. H. Takasugi, T. Takeshita, Y. Takubo, M. Talby, A. A. Talyshev, J. Tanaka, M. Tanaka, R. Tanaka, S. Tanaka, R. Tanioka, B. B. Tannenwald, S. Tapia Araya, S. Tapprogge, S. Tarem, G. F. Tartarelli, P. Tas, M. Tasevsky, T. Tashiro, E. Tassi, A. Tavares Delgado, Y. Tayalati, A. C. Taylor, A. J. Taylor, G. N. Taylor, P. T. E. Taylor, W. Taylor, P. Teixeira-Dias, D. Temple, H. Ten Kate, P. K. Teng, J. J. Teoh, F. Tepel, S. Terada, K. Terashi, J. Terron, S. Terzo, M. Testa, R. J. Teuscher, T. Theveneaux-Pelzer, F. Thiele, J. P. Thomas, J. Thomas-Wilsker, P. D. Thompson, A. S. Thompson, L. A. Thomsen, E. Thomson, M. J. Tibbetts, R. E. Ticse Torres, V. O. Tikhomirov, Yu. A. Tikhonov, S. Timoshenko, P. Tipton, S. Tisserant, K. Todome, S. Todorova-Nova, S. Todt, J. Tojo, S. Tokár, K. Tokushuku, E. Tolley, L. Tomlinson, M. Tomoto, L. Tompkins, K. Toms, B. Tong, P. Tornambe, E. Torrence, H. Torres, E. Torró Pastor, J. Toth, F. Touchard, D. R. Tovey, C. J. Treado, T. Trefzger, F. Tresoldi, A. Tricoli, I. M. Trigger, S. Trincaz-Duvoid, M. F. Tripiana, W. Trischuk, B. Trocmé, A. Trofymov, C. Troncon, M. Trottier-McDonald, M. Trovatelli, L. Truong, M. Trzebinski, A. Trzupek, K. W. Tsang, J. C-L. Tseng, P. V. Tsiareshka, G. Tsipolitis, N. Tsirintanis, S. Tsiskaridze, V. Tsiskaridze, E. G. Tskhadadze, K. M. Tsui, I. I. Tsukerman, V. Tsulaia, S. Tsuno, D. Tsybychev, Y. Tu, A. Tudorache, V. Tudorache, T. T. Tulbure, A. N. Tuna, S. A. Tupputi, S. Turchikhin, D. Turgeman, I. Turk Cakir, R. Turra, P. M. Tuts, G. Ucchielli, I. Ueda, M. Ughetto, F. Ukegawa, G. Unal, A. Undrus, G. Unel, F. C. Ungaro, Y. Unno, C. Unverdorben, J. Urban, P. Urquijo, P. Urrejola, G. Usai, J. Usui, L. Vacavant, V. Vacek, B. Vachon, K. O. H. Vadla, A. Vaidya, C. Valderanis, E. Valdes Santurio, M. Valente, S. Valentinetti, A. Valero, L. Valéry, S. Valkar, A. Vallier, J. A. Valls Ferrer, W. Van Den Wollenberg, H. van der Graaf, P. van Gemmeren, J. Van Nieuwkoop, I. van Vulpen, M. C. van Woerden, M. Vanadia, W. Vandelli, A. Vaniachine, P. Vankov, G. Vardanyan, R. Vari, E. W. Varnes, C. Varni, T. Varol, D. Varouchas, A. Vartapetian, K. E. Varvell, J. G. Vasquez, G. A. Vasquez, F. Vazeille, D. Vazquez Furelos, T. Vazquez Schroeder, J. Veatch, V. Veeraraghavan, L. M. Veloce, F. Veloso, S. Veneziano, A. Ventura, M. Venturi, N. Venturi, A. Venturini, V. Vercesi, M. Verducci, W. Verkerke, A. T. Vermeulen, J. C. Vermeulen, M. C. Vetterli, N. Viaux Maira, O. Viazlo, I. Vichou, T. Vickey, O. E. Vickey Boeriu, G. H. A. Viehhauser, S. Viel, L. Vigani, M. Villa, M. Villaplana Perez, E. Vilucchi, M. G. Vincter, V. B. Vinogradov, A. Vishwakarma, C. Vittori, I. Vivarelli, S. Vlachos, M. Vogel, P. Vokac, G. Volpi, H. von der Schmitt, E. von Toerne, V. Vorobel, K. Vorobev, M. Vos, R. Voss, J. H. Vossebeld, N. Vranjes, M. Vranjes Milosavljevic, V. Vrba, M. Vreeswijk, R. Vuillermet, I. Vukotic, P. Wagner, W. Wagner, J. Wagner-Kuhr, H. Wahlberg, S. Wahrmund, J. Walder, R. Walker, W. Walkowiak, V. Wallangen, C. Wang, C. Wang, F. Wang, H. Wang, H. Wang, J. Wang, J. Wang, Q. Wang, R. Wang, S. M. Wang, T. Wang, W. Wang, W. Wang, Z. Wang, C. Wanotayaroj, A. Warburton, C. P. Ward, D. R. Wardrope, A. Washbrook, P. M. Watkins, A. T. Watson, M. F. Watson, G. Watts, S. Watts, B. M. Waugh, A. F. Webb, S. Webb, M. S. Weber, S. W. Weber, S. A. Weber, J. S. Webster, A. R. Weidberg, B. Weinert, J. Weingarten, M. Weirich, C. Weiser, H. Weits, P. S. Wells, T. Wenaus, T. Wengler, S. Wenig, N. Wermes, M. D. Werner, P. Werner, M. Wessels, T. D. Weston, K. Whalen, N. L. Whallon, A. M. Wharton, A. S. White, A. White, M. J. White, R. White, D. Whiteson, B. W. Whitmore, F. J. Wickens, W. Wiedenmann, M. Wielers, C. Wiglesworth, L. A. M. Wiik-Fuchs, A. Wildauer, F. Wilk, H. G. Wilkens, H. H. Williams, S. Williams, C. Willis, S. Willocq, J. A. Wilson, I. Wingerter-Seez, E. Winkels, F. Winklmeier, O. J. Winston, B. T. Winter, M. Wittgen, M. Wobisch, T. M. H. Wolf, R. Wolff, M. W. Wolter, H. Wolters, V. W. S. Wong, S. D. Worm, B. K. Wosiek, J. Wotschack, K. W. Wozniak, M. Wu, S. L. Wu, X. Wu, Y. Wu, T. R. Wyatt, B. M. Wynne, S. Xella, Z. Xi, L. Xia, D. Xu, L. Xu, T. Xu, B. Yabsley, S. Yacoob, D. Yamaguchi, Y. Yamaguchi, A. Yamamoto, S. Yamamoto, T. Yamanaka, F. Yamane, M. Yamatani, Y. Yamazaki, Z. Yan, H. Yang, H. Yang, Y. Yang, Z. Yang, W-M. Yao, Y. C. Yap, Y. Yasu, E. Yatsenko, K. H. Yau Wong, J. Ye, S. Ye, I. Yeletskikh, E. Yigitbasi, E. Yildirim, K. Yorita, K. Yoshihara, C. Young, C. J. S. Young, J. Yu, J. Yu, S. P. Y. Yuen, I. Yusuff, B. Zabinski, G. Zacharis, R. Zaidan, A. M. Zaitsev, N. Zakharchuk, J. Zalieckas, A. Zaman, S. Zambito, D. Zanzi, C. Zeitnitz, G. Zemaityte, A. Zemla, J. C. Zeng, Q. Zeng, O. Zenin, T. Ženiš, D. Zerwas, D. Zhang, F. Zhang, G. Zhang, H. Zhang, J. Zhang, L. Zhang, L. Zhang, M. Zhang, P. Zhang, R. Zhang, R. Zhang, X. Zhang, Y. Zhang, Z. Zhang, X. Zhao, Y. Zhao, Z. Zhao, A. Zhemchugov, B. Zhou, C. Zhou, L. Zhou, M. Zhou, M. Zhou, N. Zhou, C. G. Zhu, H. Zhu, J. Zhu, Y. Zhu, X. Zhuang, K. Zhukov, A. Zibell, D. Zieminska, N. I. Zimine, C. Zimmermann, S. Zimmermann, Z. Zinonos, M. Zinser, M. Ziolkowski, L. Živković, G. Zobernig, A. Zoccoli, R. Zou, M. zur Nedden, L. Zwalinski, ATLAS Collaboration

**Affiliations:** 1 Department of Physics, University of Adelaide, Adelaide, Australia; 2 Physics Department, SUNY Albany, Albany, NY USA; 3 Department of Physics, University of Alberta, Edmonton, AB Canada; 4 Department of Physics, Ankara University, Ankara, Turkey; 5 Istanbul Aydin University, Istanbul, Turkey; 6 Division of Physics, TOBB University of Economics and Technology, Ankara, Turkey; 7 LAPP, CNRS/IN2P3 and Université Savoie Mont Blanc, Annecy-le-Vieux, France; 8 High Energy Physics Division, Argonne National Laboratory, Argonne, IL USA; 9 Department of Physics, University of Arizona, Tucson, AZ USA; 10 Department of Physics, The University of Texas at Arlington, Arlington, TX USA; 11 Physics Department, National and Kapodistrian University of Athens, Athens, Greece; 12 Physics Department, National Technical University of Athens, Zografou, Greece; 13 Department of Physics, The University of Texas at Austin, Austin, TX USA; 14 Institute of Physics, Azerbaijan Academy of Sciences, Baku, Azerbaijan; 15 Institut de Física d’Altes Energies (IFAE), The Barcelona Institute of Science and Technology, Barcelona, Spain; 16 Institute of Physics, University of Belgrade, Belgrade, Serbia; 17 Department for Physics and Technology, University of Bergen, Bergen, Norway; 18 Physics Division, Lawrence Berkeley National Laboratory, University of California, Berkeley, CA USA; 19 Department of Physics, Humboldt University, Berlin, Germany; 20 Albert Einstein Center for Fundamental Physics, Laboratory for High Energy Physics, University of Bern, Bern, Switzerland; 21 School of Physics and Astronomy, University of Birmingham, Birmingham, UK; 22 Department of Physics, Bogazici University, Istanbul, Turkey; 23 Department of Physics Engineering, Gaziantep University, Gaziantep, Turkey; 24 Faculty of Engineering and Natural Sciences, Istanbul Bilgi University, Istanbul, Turkey; 25 Faculty of Engineering and Natural Sciences, Bahcesehir University, Istanbul, Turkey; 26 Centro de Investigaciones, Universidad Antonio Narino, Bogota, Colombia; 27 INFN Sezione di Bologna, Bologna, Italy; 28 Dipartimento di Fisica e Astronomia, Università di Bologna, Bologna, Italy; 29 Physikalisches Institut, University of Bonn, Bonn, Germany; 30 Department of Physics, Boston University, Boston, MA USA; 31 Department of Physics, Brandeis University, Waltham, MA USA; 32 Universidade Federal do Rio De Janeiro COPPE/EE/IF, Rio de Janeiro, Brazil; 33 Electrical Circuits Department, Federal University of Juiz de Fora (UFJF), Juiz de Fora, Brazil; 34 Federal University of Sao Joao del Rei (UFSJ), Sao Joao del Rei, Brazil; 35 Instituto de Fisica, Universidade de Sao Paulo, São Paulo, Brazil; 36 Physics Department, Brookhaven National Laboratory, Upton, NY USA; 37 Transilvania University of Brasov, Brasov, Romania; 38 Horia Hulubei National Institute of Physics and Nuclear Engineering, Bucharest, Romania; 39 Department of Physics, Alexandru Ioan Cuza University of Iasi, Iasi, Romania; 40 Physics Department, National Institute for Research and Development of Isotopic and Molecular Technologies, Cluj-Napoca, Romania; 41 University Politehnica Bucharest, Bucharest, Romania; 42 West University in Timisoara, Timisoara, Romania; 43 Departamento de Física, Universidad de Buenos Aires, Buenos Aires, Argentina; 44 Cavendish Laboratory, University of Cambridge, Cambridge, UK; 45 Department of Physics, Carleton University, Ottawa, ON Canada; 46 CERN, Geneva, Switzerland; 47 Enrico Fermi Institute, University of Chicago, Chicago, IL USA; 48 Departamento de Física, Pontificia Universidad Católica de Chile, Santiago, Chile; 49 Departamento de Física, Universidad Técnica Federico Santa María, Valparaíso, Chile; 50 Institute of High Energy Physics, Chinese Academy of Sciences, Beijing, China; 51 Department of Physics, Nanjing University, Nanjing, Jiangsu China; 52 Physics Department, Tsinghua University, Beijing, 100084 China; 53 University of Chinese Academy of Science (UCAS), Beijing, China; 54 Department of Modern Physics and State Key Laboratory of Particle Detection and Electronics, University of Science and Technology of China, Hefei, Anhui China; 55 School of Physics, Shandong University, Jinan, Shandong China; 56 Department of Physics and Astronomy, Key Laboratory for Particle Physics, Astrophysics and Cosmology, Ministry of Education, Shanghai Key Laboratory for Particle Physics and Cosmology, Shanghai Jiao Tong University, Shanghai (also at PKU-CHEP), Shanghai, China; 57 Université Clermont Auvergne, CNRS/IN2P3, LPC, Clermont-Ferrand, France; 58 Nevis Laboratory, Columbia University, Irvington, NY USA; 59 Niels Bohr Institute, University of Copenhagen, Copenhagen, Denmark; 60 INFN Gruppo Collegato di Cosenza, Laboratori Nazionali di Frascati, Frascati, Italy; 61 Dipartimento di Fisica, Università della Calabria, Rende, Italy; 62 Faculty of Physics and Applied Computer Science, AGH University of Science and Technology, Kraków, Poland; 63 Marian Smoluchowski Institute of Physics, Jagiellonian University, Kraków, Poland; 64 Institute of Nuclear Physics, Polish Academy of Sciences, Kraków, Poland; 65 Physics Department, Southern Methodist University, Dallas, TX USA; 66 Physics Department, University of Texas at Dallas, Richardson, TX USA; 67 DESY, Hamburg and Zeuthen, Germany; 68 Lehrstuhl für Experimentelle Physik IV, Technische Universität Dortmund, Dortmund, Germany; 69 Institut für Kern- und Teilchenphysik, Technische Universität Dresden, Dresden, Germany; 70 Department of Physics, Duke University, Durham, NC USA; 71 SUPA-School of Physics and Astronomy, University of Edinburgh, Edinburgh, UK; 72 INFN e Laboratori Nazionali di Frascati, Frascati, Italy; 73 Fakultät für Mathematik und Physik, Albert-Ludwigs-Universität, Freiburg, Germany; 74 Departement de Physique Nucleaire et Corpusculaire, Université de Genève, Geneva, Switzerland; 75 INFN Sezione di Genova, Genoa, Italy; 76 Dipartimento di Fisica, Università di Genova, Genoa, Italy; 77 E. Andronikashvili Institute of Physics, Iv. Javakhishvili Tbilisi State University, Tbilisi, Georgia; 78 High Energy Physics Institute, Tbilisi State University, Tbilisi, Georgia; 79 II Physikalisches Institut, Justus-Liebig-Universität Giessen, Giessen, Germany; 80 SUPA-School of Physics and Astronomy, University of Glasgow, Glasgow, UK; 81 II Physikalisches Institut, Georg-August-Universität, Göttingen, Germany; 82 Laboratoire de Physique Subatomique et de Cosmologie, Université Grenoble-Alpes, CNRS/IN2P3, Grenoble, France; 83 Laboratory for Particle Physics and Cosmology, Harvard University, Cambridge, MA USA; 84 Kirchhoff-Institut für Physik, Ruprecht-Karls-Universität Heidelberg, Heidelberg, Germany; 85 Physikalisches Institut, Ruprecht-Karls-Universität Heidelberg, Heidelberg, Germany; 86 Faculty of Applied Information Science, Hiroshima Institute of Technology, Hiroshima, Japan; 87 Department of Physics, The Chinese University of Hong Kong, Shatin, NT Hong Kong; 88 Department of Physics, The University of Hong Kong, Hong Kong, China; 89 Department of Physics, Institute for Advanced Study, The Hong Kong University of Science and Technology, Clear Water Bay, Kowloon, Hong Kong, China; 90 Department of Physics, National Tsing Hua University, Hsinchu, Taiwan; 91 Department of Physics, Indiana University, Bloomington, IN USA; 92 Institut für Astro- und Teilchenphysik, Leopold-Franzens-Universität, Innsbruck, Austria; 93 University of Iowa, Iowa City, IA USA; 94 Department of Physics and Astronomy, Iowa State University, Ames, IA USA; 95 Joint Institute for Nuclear Research, JINR Dubna, Dubna, Russia; 96 KEK, High Energy Accelerator Research Organization, Tsukuba, Japan; 97 Graduate School of Science, Kobe University, Kobe, Japan; 98 Faculty of Science, Kyoto University, Kyoto, Japan; 99 Kyoto University of Education, Kyoto, Japan; 100 Research Center for Advanced Particle Physics and Department of Physics, Kyushu University, Fukuoka, Japan; 101 Instituto de Física La Plata, Universidad Nacional de La Plata and CONICET, La Plata, Argentina; 102 Physics Department, Lancaster University, Lancaster, UK; 103 INFN Sezione di Lecce, Lecce, Italy; 104 Dipartimento di Matematica e Fisica, Università del Salento, Lecce, Italy; 105 Oliver Lodge Laboratory, University of Liverpool, Liverpool, UK; 106 Department of Experimental Particle Physics, Jožef Stefan Institute and Department of Physics, University of Ljubljana, Ljubljana, Slovenia; 107 School of Physics and Astronomy, Queen Mary University of London, London, UK; 108 Department of Physics, Royal Holloway University of London, Surrey, UK; 109 Department of Physics and Astronomy, University College London, London, UK; 110 Louisiana Tech University, Ruston, LA USA; 111 Laboratoire de Physique Nucléaire et de Hautes Energies, UPMC and Université Paris-Diderot and CNRS/IN2P3, Paris, France; 112 Fysiska institutionen, Lunds universitet, Lund, Sweden; 113 Departamento de Fisica Teorica C-15, Universidad Autonoma de Madrid, Madrid, Spain; 114 Institut für Physik, Universität Mainz, Mainz, Germany; 115 School of Physics and Astronomy, University of Manchester, Manchester, UK; 116 CPPM, Aix-Marseille Université and CNRS/IN2P3, Marseille, France; 117 Department of Physics, University of Massachusetts, Amherst, MA USA; 118 Department of Physics, McGill University, Montreal, QC Canada; 119 School of Physics, University of Melbourne, Victoria, Australia; 120 Department of Physics, The University of Michigan, Ann Arbor, MI USA; 121 Department of Physics and Astronomy, Michigan State University, East Lansing, MI USA; 122 INFN Sezione di Milano, Milan, Italy; 123 Dipartimento di Fisica, Università di Milano, Milan, Italy; 124 B.I. Stepanov Institute of Physics, National Academy of Sciences of Belarus, Minsk, Republic of Belarus; 125 Research Institute for Nuclear Problems of Byelorussian State University, Minsk, Republic of Belarus; 126 Group of Particle Physics, University of Montreal, Montreal, QC Canada; 127 P.N. Lebedev Physical Institute of the Russian Academy of Sciences, Moscow, Russia; 128 Institute for Theoretical and Experimental Physics (ITEP), Moscow, Russia; 129 National Research Nuclear University MEPhI, Moscow, Russia; 130 D.V. Skobeltsyn Institute of Nuclear Physics, M.V. Lomonosov Moscow State University, Moscow, Russia; 131 Fakultät für Physik, Ludwig-Maximilians-Universität München, Munich, Germany; 132 Max-Planck-Institut für Physik (Werner-Heisenberg-Institut), Munich, Germany; 133 Nagasaki Institute of Applied Science, Nagasaki, Japan; 134 Graduate School of Science and Kobayashi-Maskawa Institute, Nagoya University, Nagoya, Japan; 135 INFN Sezione di Napoli, Naples, Italy; 136 Dipartimento di Fisica, Università di Napoli, Naples, Italy; 137 Department of Physics and Astronomy, University of New Mexico, Albuquerque, NM USA; 138 Institute for Mathematics, Astrophysics and Particle Physics, Radboud University Nijmegen/Nikhef, Nijmegen, The Netherlands; 139 Nikhef National Institute for Subatomic Physics, University of Amsterdam, Amsterdam, The Netherlands; 140 Department of Physics, Northern Illinois University, DeKalb, IL USA; 141 Budker Institute of Nuclear Physics, SB RAS, Novosibirsk, Russia; 142 Department of Physics, New York University, New York, NY USA; 143 Ohio State University, Columbus, OH USA; 144 Faculty of Science, Okayama University, Okayama, Japan; 145 Homer L. Dodge Department of Physics and Astronomy, University of Oklahoma, Norman, OK USA; 146 Department of Physics, Oklahoma State University, Stillwater, OK USA; 147 Palacký University, RCPTM, Olomouc, Czech Republic; 148 Center for High Energy Physics, University of Oregon, Eugene, OR USA; 149 LAL, Univ. Paris-Sud, CNRS/IN2P3, Université Paris-Saclay, Orsay, France; 150 Graduate School of Science, Osaka University, Osaka, Japan; 151 Department of Physics, University of Oslo, Oslo, Norway; 152 Department of Physics, Oxford University, Oxford, UK; 153 INFN Sezione di Pavia, Pavia, Italy; 154 Dipartimento di Fisica, Università di Pavia, Pavia, Italy; 155 Department of Physics, University of Pennsylvania, Philadelphia, PA USA; 156 National Research Centre “Kurchatov Institute” B.P. Konstantinov Petersburg Nuclear Physics Institute, St. Petersburg, Russia; 157 INFN Sezione di Pisa, Pisa, Italy; 158 Dipartimento di Fisica E. Fermi, Università di Pisa, Pisa, Italy; 159 Department of Physics and Astronomy, University of Pittsburgh, Pittsburgh, PA USA; 160 Laboratório de Instrumentação e Física Experimental de Partículas-LIP, Lisbon, Portugal; 161 Faculdade de Ciências, Universidade de Lisboa, Lisbon, Portugal; 162 Department of Physics, University of Coimbra, Coimbra, Portugal; 163 Centro de Física Nuclear da Universidade de Lisboa, Lisbon, Portugal; 164 Departamento de Fisica, Universidade do Minho, Braga, Portugal; 165 Departamento de Fisica Teorica y del Cosmos, Universidad de Granada, Granada, Spain; 166 Dep Fisica and CEFITEC of Faculdade de Ciencias e Tecnologia, Universidade Nova de Lisboa, Caparica, Portugal; 167 Institute of Physics, Academy of Sciences of the Czech Republic, Prague, Czech Republic; 168 Czech Technical University in Prague, Prague, Czech Republic; 169 Faculty of Mathematics and Physics, Charles University, Prague, Czech Republic; 170 State Research Center Institute for High Energy Physics (Protvino), NRC KI, Protvino, Russia; 171 Particle Physics Department, Rutherford Appleton Laboratory, Didcot, UK; 172 INFN Sezione di Roma, Rome, Italy; 173 Dipartimento di Fisica, Sapienza Università di Roma, Rome, Italy; 174 INFN Sezione di Roma Tor Vergata, Rome, Italy; 175 Dipartimento di Fisica, Università di Roma Tor Vergata, Rome, Italy; 176 INFN Sezione di Roma Tre, Rome, Italy; 177 Dipartimento di Matematica e Fisica, Università Roma Tre, Rome, Italy; 178 Faculté des Sciences Ain Chock, Réseau Universitaire de Physique des Hautes Energies-Université Hassan II, Casablanca, Morocco; 179 Centre National de l’Energie des Sciences Techniques Nucleaires, Rabat, Morocco; 180 Faculté des Sciences Semlalia, Université Cadi Ayyad, LPHEA-Marrakech, Marrakech, Morocco; 181 Faculté des Sciences, Université Mohamed Premier and LPTPM, Oujda, Morocco; 182 Faculté des Sciences, Université Mohammed V, Rabat, Morocco; 183 DSM/IRFU (Institut de Recherches sur les Lois Fondamentales de l’Univers), CEA Saclay (Commissariat à l’Energie Atomique et aux Energies Alternatives), Gif-sur-Yvette, France; 184 Santa Cruz Institute for Particle Physics, University of California Santa Cruz, Santa Cruz, CA USA; 185 Department of Physics, University of Washington, Seattle, WA USA; 186 Department of Physics and Astronomy, University of Sheffield, Sheffield, UK; 187 Department of Physics, Shinshu University, Nagano, Japan; 188 Department Physik, Universität Siegen, Siegen, Germany; 189 Department of Physics, Simon Fraser University, Burnaby, BC Canada; 190 SLAC National Accelerator Laboratory, Stanford, CA USA; 191 Faculty of Mathematics, Physics and Informatics, Comenius University, Bratislava, Slovak Republic; 192 Department of Subnuclear Physics, Institute of Experimental Physics of the Slovak Academy of Sciences, Kosice, Slovak Republic; 193 Department of Physics, University of Cape Town, Cape Town, South Africa; 194 Department of Physics, University of Johannesburg, Johannesburg, South Africa; 195 School of Physics, University of the Witwatersrand, Johannesburg, South Africa; 196 Department of Physics, Stockholm University, Stockholm, Sweden; 197 The Oskar Klein Centre, Stockholm, Sweden; 198 Physics Department, Royal Institute of Technology, Stockholm, Sweden; 199 Departments of Physics and Astronomy and Chemistry, Stony Brook University, Stony Brook, NY USA; 200 Department of Physics and Astronomy, University of Sussex, Brighton, UK; 201 School of Physics, University of Sydney, Sydney, Australia; 202 Institute of Physics, Academia Sinica, Taipei, Taiwan; 203 Department of Physics, Technion: Israel Institute of Technology, Haifa, Israel; 204 Raymond and Beverly Sackler School of Physics and Astronomy, Tel Aviv University, Tel Aviv, Israel; 205 Department of Physics, Aristotle University of Thessaloniki, Thessaloniki, Greece; 206 International Center for Elementary Particle Physics and Department of Physics, The University of Tokyo, Tokyo, Japan; 207 Graduate School of Science and Technology, Tokyo Metropolitan University, Tokyo, Japan; 208 Department of Physics, Tokyo Institute of Technology, Tokyo, Japan; 209 Tomsk State University, Tomsk, Russia; 210 Department of Physics, University of Toronto, Toronto, ON Canada; 211 INFN-TIFPA, Trento, Italy; 212 University of Trento, Trento, Italy; 213 TRIUMF, Vancouver, BC Canada; 214 Department of Physics and Astronomy, York University, Toronto, ON Canada; 215 Faculty of Pure and Applied Sciences, and Center for Integrated Research in Fundamental Science and Engineering, University of Tsukuba, Tsukuba, Japan; 216 Department of Physics and Astronomy, Tufts University, Medford, MA USA; 217 Department of Physics and Astronomy, University of California Irvine, Irvine, CA USA; 218 INFN Gruppo Collegato di Udine, Sezione di Trieste, Udine, Italy; 219 ICTP, Trieste, Italy; 220 Dipartimento di Chimica, Fisica e Ambiente, Università di Udine, Udine, Italy; 221 Department of Physics and Astronomy, University of Uppsala, Uppsala, Sweden; 222 Department of Physics, University of Illinois, Urbana, IL USA; 223 Instituto de Fisica Corpuscular (IFIC), Centro Mixto Universidad de Valencia-CSIC, Valencia, Spain; 224 Department of Physics, University of British Columbia, Vancouver, BC Canada; 225 Department of Physics and Astronomy, University of Victoria, Victoria, BC Canada; 226 Department of Physics, University of Warwick, Coventry, UK; 227 Waseda University, Tokyo, Japan; 228 Department of Particle Physics, The Weizmann Institute of Science, Rehovot, Israel; 229 Department of Physics, University of Wisconsin, Madison, WI USA; 230 Fakultät für Physik und Astronomie, Julius-Maximilians-Universität, Würzburg, Germany; 231 Fakultät für Mathematik und Naturwissenschaften, Fachgruppe Physik, Bergische Universität Wuppertal, Wuppertal, Germany; 232 Department of Physics, Yale University, New Haven, CT USA; 233 Yerevan Physics Institute, Yerevan, Armenia; 234 Centre de Calcul de l’Institut National de Physique Nucléaire et de Physique des Particules (IN2P3), Villeurbanne, France; 235 Academia Sinica Grid Computing, Institute of Physics, Academia Sinica, Taipei, Taiwan; 236 CERN, 1211 Geneva 23, Switzerland

## Abstract

A search for the direct production of charginos and neutralinos in final states with at least two hadronically decaying tau leptons is presented. The analysis uses a dataset of *pp* collisions corresponding to an integrated luminosity of 36.1 fb$^{-1}$, recorded with the ATLAS detector at the Large Hadron Collider at a centre-of-mass energy of 13 TeV. No significant deviation from the expected Standard Model background is observed. Limits are derived in scenarios of  pair production and of  and  production in simplified models where the neutralinos and charginos decay solely via intermediate left-handed staus and tau sneutrinos, and the mass of the $\tilde{\tau }_{\mathrm L}$ state is set to be halfway between the masses of the  and the . Chargino masses up to 630 GeV are excluded at 95% confidence level in the scenario of direct production of  for a massless . Common  and  masses up to 760 GeV are excluded in the case of production of  and  assuming a massless . Exclusion limits for additional benchmark scenarios with large and small mass-splitting between the  and the  are also studied by varying the $\tilde{\tau }_{\mathrm L}$ mass between the masses of the  and the .

## Introduction

Supersymmetry (SUSY) [1–7] postulates the existence of a superpartner, referred to as a *sparticle*, whose spin differs by one half unit from each corresponding Standard Model (SM) partner. In models that conserve *R*-parity [8], sparticles are always produced in pairs, and the lightest supersymmetric particle (LSP) is stable and provides a dark-matter candidate [9–11].

In SUSY models, the sector of sparticles with only electroweak interactions contains charginos ($\tilde{\chi } ^{\pm }_{i}$, *i* = 1, 2 in order of increasing masses), neutralinos ($\tilde{\chi } ^{0}_{j}$, *j* = 1, 2, 3, 4 in order of increasing masses), sleptons ($\tilde{\ell }$), and sneutrinos ($\tilde{\nu }$). Charginos and neutralinos are the mass eigenstates formed from the linear superpositions of the superpartners of the charged and neutral Higgs bosons and electroweak gauge bosons. The sleptons are the superpartners of the leptons and are referred to as left or right ($\tilde{\ell }_{\mathrm {L}}$ or $\tilde{\ell }_{\mathrm {R}}$) depending on the chirality of their SM partners. The slepton mass eigenstates are a mixture of $\tilde{\ell }_{\mathrm {L}}$ and $\tilde{\ell }_{\mathrm {R}}$, and are labelled as $\tilde{\ell } _1$ and $\tilde{\ell } _2$ (with $\tilde{\ell } _{k}$, *k* = 1, 2 in order of increasing masses). In this work, only the , the , the , and the scalar superpartner of the left-handed tau lepton (the stau, $\tilde{\tau }_{\mathrm {L}}$) and of the tau neutrino (the tau sneutrino, $\tilde{\nu } _{\tau _L}$) are assumed to be sufficiently light to be produced at the Large Hadron Collider (LHC) [12].

Although experimentally challenging, final states with tau leptons originating from stau decays are of particular interest for SUSY searches. Models with light staus can lead to a dark-matter relic density consistent with cosmological observations [13], and light sleptons in general could play a role in the co-annihilation of neutralinos [14, 15]. Sleptons are expected to have masses of $\mathcal {O}$(100 GeV) in gauge-mediated [16–21] and anomaly-mediated [22, 23] SUSY breaking models.

**Fig. 1:**
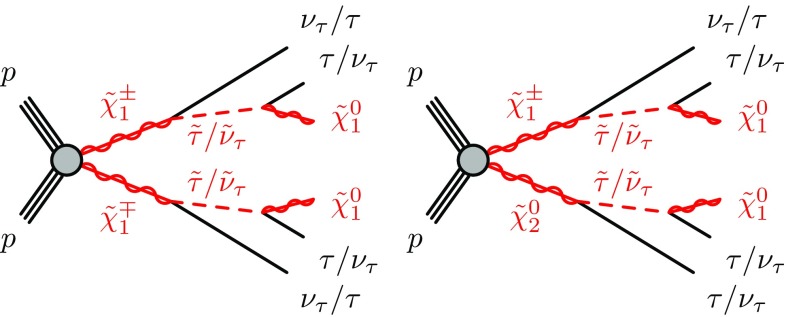
Representative diagrams for the electroweak production and decay processes of supersymmetric particles considered in this work: (left)  and (right)  production

Scenarios where the production of charginos, neutralinos, and sleptons may dominate at the LHC with respect to the production of squarks and gluinos can be realised in the general framework of the phenomenological Minimal Supersymmetric Standard Model (pMSSM) [24, 25]. Two simplified models [26–28] of  and  production are considered in this work. The models are designed to enhance the probability of experimental observation. In both models, the lightest neutralino is the LSP and purely bino, the stau and tau sneutrino are assumed to be mass-degenerate, and the $\tilde{\tau } _1$ is assumed to be purely $\tilde{\tau }_{\mathrm {L}}$. The mass of the $\tilde{\tau }_{\mathrm L}$ state is set to be halfway between the masses of the  and the , i.e. , with the parameter $x=0.5$. Other values of *x* are also studied for selected benchmark models where *x* is varied between 0.05 and 0.95 in steps of 0.1. All sparticles other than those explicitely mentioned here are assumed to be inaccessible at the LHC energy. In the model characterised by  production, the  and  are assumed to be pure wino and mass-degenerate. In the model where only  production is considered, the  is pure wino. The above assumptions guarantee large production cross sections and short decay chains for  and . Charginos and next-to-lightest neutralinos decay into the lightest neutralino via an intermediate on-shell stau or tau sneutrino, , , and  (see Fig. 1).

Signal events are characterised by the presence of at least two tau leptons and large missing transverse energy, $E_{\text {T}}^{\text {miss}}$, due to the undetected neutrinos and lightest neutralinos. Final states with at least two hadronically decaying tau leptons (tau $\rightarrow$ hadrons $\nu _\tau$) are considered, as this choice provides the best discrimination of SUSY events of interest from SM background processes (mainly multi-jet, *W* + jets and diboson production). In multi-jet events passing the selection requirements described in Sect. 5, nearly all reconstructed tau leptons are misidentified jets. *W*($\rightarrow \tau \nu _\tau$) + jets events contribute due to the $E_{\text {T}}^{\text {miss}}$ from the neutrino, one tau lepton from the *W* decay, and one or more jets misidentified as tau leptons. The jet misidentification typically results in a mismeasurement of $E_{\text {T}}^{\text {miss}}$, which tends to assume large values. Diboson events with *WW* or *ZZ* decaying into $\tau \tau \nu \nu$ final states contain two tau leptons and large $E_{\text {T}}^{\text {miss}}$ from the neutrinos.

The search described in this paper uses a dataset of $\sqrt{s}=13$ TeV *pp* collisions collected with the ATLAS detector in 2015 and 2016, with an integrated luminosity of 36.1 fb$^{-1}$. In a previous similar search by the ATLAS Collaboration using the 8 TeV Run-1 dataset [29],  masses up to 345 GeV were excluded at 95% confidence level for a massless  in the scenario of direct production of . In the case of production of  and , common  and  masses up to 410 GeV were excluded for a massless . Results of a similar search in the Run-1 dataset from the CMS Collaboration are reported in Refs. [30, 31]. In Ref. [30], charginos lighter than 320 GeV are excluded at 95% confidence level in the case of a massless . The combined LEP limits on the stau and chargino^1^ masses are $m_{\widetilde{\tau }}>87$–$93\mathrm \,GeV$ (depending on ) and  [32–36], respectively.

## ATLAS detector

The ATLAS detector [37] is a multi-purpose particle physics detector with forward-backward symmetric cylindrical geometry and nearly $4\pi$ coverage in solid angle.^2^ It features an inner tracking detector (ID) surrounded by a 2 T superconducting solenoid, electromagnetic and hadronic calorimeters, and a muon spectrometer (MS). The ID covers the pseudorapidity region $|\eta | <2.5$ and consists of a silicon pixel detector, a silicon microstrip detector, and a transition radiation tracker. One significant upgrade for the $\sqrt{s} = 13\,\mathrm{TeV}$ running period is the presence of the insertable B-Layer [38], an additional pixel layer close to the interaction point which provides high-resolution hits at small radius to improve the tracking and vertex reconstruction performance. The calorimeters are composed of high-granularity liquid-argon (LAr) electromagnetic calorimeters with lead, copper, or tungsten absorbers (in the pseudorapidity region $|\eta | < 3.2$) and a steel–scintillator hadronic calorimeter (for $|\eta |<1.7$). The end-cap and forward regions, spanning $1.5<|\eta |<4.9$, are instrumented with LAr calorimeters for both the electromagnetic and hadronic measurements. The MS surrounds the calorimeters and consists of three large superconducting air-core toroidal magnets, each with eight coils, a system of precision tracking chambers ($|\eta |<2.7$), and detectors for triggering ($|\eta |<2.4$). A two-level trigger system is used to record events [39].

## Data and simulated event samples

The analysed dataset, after the application of beam, detector, and data quality requirements, corresponds to an integrated luminosity of 36.1 fb$^{-1}$of *pp* collision data recorded in 2015 and 2016 at $\sqrt{s} = 13\,\mathrm{TeV}$. The uncertainty in the combined 2015 + 2016 integrated luminosity is 3.2%. It is derived, following a methodology similar to that detailed in Ref. [40], from a preliminary calibration of the luminosity scale using $x-y$ beam-separation scans performed in August 2015 and May 2016.

Monte Carlo (MC) simulated event samples are used to estimate the SUSY signal yields and to aid in evaluating the SM backgrounds. Generated SM events are processed through a detailed detector simulation [41] based on Geant 4 [42], whereas SUSY events are passed through a fast detector simulation based on a parameterisation of the performance of the ATLAS electromagnetic and hadronic calorimeters [43] and Geant 4 elsewhere. All simulated events are overlaid with multiple *pp* collisions (pile-up) simulated with the soft strong interaction processes of Pythia 8.186 [44] using the A2 set of tuned parameters [45] and the MSTW2008LO [46] PDF set. The simulated events are reconstructed using the same algorithms as the data, and are reweighted so that the distribution of the expected number of collisions per bunch crossing matches the one in the data.

### Simulated background samples

Events with $Z/\gamma ^*\rightarrow \ell \ell$ $(\ell =e,\mu ,\tau )$ and $W\rightarrow \ell \nu$ produced with accompanying jets (including light and heavy flavours) were generated at next-to-leading order (NLO) in the strong coupling constant with Sherpa 2.2.0 and 2.2.1 [47, 48]. Matrix elements (ME) were calculated for up to two additional partons at NLO and four additional partons at leading order (LO), using the Comix [49] and OpenLoops [50] generators and merged with the Sherpa parton shower (PS) [51] using the ME + PS@NLO prescription [48]. The NNPDF3.0NNLO [52] parton distribution function (PDF) set was used in conjunction with a dedicated parton-shower tuning developed by the Sherpa authors. The *W* / *Z* + jets events were normalised using their next-to-next-to-leading order (NNLO) cross sections [53]. For Sherpa 2.2.0 samples, a simplified scale setting prescription was used in the multi-parton matrix elements, to improve the event generation speed.

The fully leptonic diboson processes ($VV=WW/WZ/ZZ$) were generated using Sherpa 2.2.1 including final states with all possible combinations of charged leptons and neutrinos. The matrix elements contain all diagrams with four electroweak vertices, and they were calculated for up to one parton ($4 \ell$, $2 \ell$ + $2\nu$, *ZZ*, *WW*) or no additional parton ($3 \ell$+$1 \nu$, $1 \ell$ + $3 \nu$, *WZ*) at NLO and up to three partons at LO. The NNPDF3.0NNLO PDF set was used in conjunction with a dedicated PS tuning developed by the Sherpa authors. Diboson processes with one of the bosons decaying hadronically and the other leptonically were simulated using the Sherpa 2.1.1 event generator. The matrix elements are calculated for up to one (*ZZ*) or no (*WW*, *WZ*) additional partons at NLO and up to three additional partons at LO. The CT10 [54] PDF set was used in conjunction with a dedicated PS tuning developed by the Sherpa authors. Each of the diboson processes was normalised using the corresponding NLO cross Sect. [55].

The production of top-quark pairs and single top quarks in the *Wt* and *s*-channels was performed with Powheg-Box 2 [56], with the CT10 PDF set in the ME calculations. Electroweak *t*-channel single-top-quark events were generated using the Powheg-Box 1 event generator. The PS, fragmentation, and the underlying event were simulated using Pythia 6.428 [57] with the CTEQ6L1 PDF set and a corresponding set of tuned parameters called the Perugia 2012 tune [58]. The EvtGen 1.2.0 program [59] was used for properties of the bottom and charm hadron decays. The top-quark mass was set to 172.5 GeV. The overall cross section was computed at NNLO in $\alpha _\mathrm {s}$, including resummation of next-to-next-to-leading-logarithm (NNLL) soft gluon terms [60] for $t\bar{t}$, to NLO + NNLL accuracy for single-top-quark *Wt*-channel [61], and to NLO for the *t*- and *s*-channels [62]. Top-quark pair production with an additional *W* or *Z* boson was performed using MadGraph5_aMC@NLO 2.2.2 [63], while fragmentation and hadronisation were simulated with Pythia 8.186. The ATLAS underlying-event tune A14 [64] was used with the NNPDF2.3LO [65] PDF set, and the cross sections were normalised using NLO [66, 67].

### Simulated signal samples

Simulated signal samples were generated using MadGraph5_aMC@NLO 2.2.3 interfaced to Pythia 8.186 with the A14 tune for the PS modelling, hadronisation, and underlying event. The ME calculation is performed at tree level and includes the emission of up to two additional partons. The PDF set used for the generation is NNPDF2.3LO. The ME–PS matching used the CKKW-L [68] prescription, with a matching scale set to one quarter of the mass of the pair of produced particles. Signal cross sections were calculated to next-to-leading order in the strong coupling constant, adding the resummation of soft gluon emission at next-to-leading-logarithm accuracy (NLO + NLL) [69, 70]. The nominal cross section and the uncertainty were taken from an envelope of cross-section predictions using different PDF sets and factorisation and renormalisation scales, following the procedure described in Ref. [71].

Two simplified models characterised by  and  production are considered. The neutralinos and charginos decay via intermediate staus and tau sneutrinos. In both models, the  mass is varied between 100 GeV and 1.1 TeV in steps of 50 (100) GeV for  masses smaller (larger) than 700 GeV. The  mass is varied between zero and 500 GeV with a variable spacing of 25 (50) GeV for  and  masses smaller (larger) than 700 and 250 GeV respectively. A total of 159 models was generated. The parameter *x* is fixed to 0.5. The cross section for  ( ) production ranges from 23 (11.6) pb for a  mass of 100 GeV to 0.74 (0.34) fb for a  mass of 1.1 TeV.

Two reference points are used throughout this paper to illustrate the typical features of the SUSY models to which this analysis is sensitive:

- Reference point 1: simplified model for  production with the masses of the  and the  equal to 600 GeV, and a massless ;
- Reference point 2: simplified model for  production with the mass of the  equal to 600 GeV, and a massless .

The dependence on the parameter *x* is evaluated in two additional scenarios for both  and  production where *x* is varied between 0.05 and 0.95 in steps of 0.1. The first benchmark model has a large mass-splitting between the  and the , with  = 600 GeV and massless , while the second model is more compressed with  = 250 GeV and  = 100 GeV.

## Event reconstruction

Events with at least one reconstructed primary vertex [72] are selected. A primary vertex must have at least two associated charged-particle tracks with transverse momentum $p_{\text {T}}>$ 400 MeV and be consistent with the beam spot envelope. If there are multiple primary vertices in an event, the one with the largest $\sum p_{\text {T}} ^2$ of the associated tracks is chosen.

Jets are reconstructed from three-dimensional calorimeter energy clusters [73] using the anti-$k_t$ algorithm [74, 75] with a radius parameter of 0.4. Jet energies are corrected for detector inhomogeneities, the non-compensating response of the calorimeter, and the impact of pile-up, using factors derived from test beam and *pp* collision data, and from a detailed Geant 4 detector simulation [76, 77]. The impact of pile-up is accounted for using a technique, based on jet areas, that provides an event-by-event and jet-by-jet correction [78]. Jets that are likely to have originated from pile-up are not considered [79]. Jets are required to have $p_{\text {T}} >20$ GeV and $\vert \eta \vert < 2.8$. Events containing jets that are likely to have arisen from detector noise or cosmic rays are removed.

Jets containing *b*-hadrons ($b\text {-jets}$) are identified using the MV2c10 algorithm, a multivariate discriminant making use of track impact parameters and reconstructed secondary vertices [80]. Candidate $b\text {-jets}$ are required to have $p_{\text {T}} > 20$ GeV and $|\eta | < 2.5$. A working point with an average *b*-tagging efficiency of 77% for simulated $t\bar{t}$ events is used [81, 82]. The expected rejection factors for light-quark and gluon jets, *c*-quark jets, and hadronically decaying tau leptons are approximately 134, 6, and 55, respectively.

Electron candidates are reconstructed by matching clusters in the electromagnetic calorimeter with charged-particle tracks in the inner detector. Electrons are required to have $p_{\text {T}} >10$ GeV, $|\eta |<2.47$, and to satisfy the ‘loose’ working point according to a likelihood-based identification [83]. Muon candidates are reconstructed from MS tracks matching ID tracks. Muons are required to have $p_{\text {T}} > 10$ GeV and $\vert \eta \vert < 2.7$ and fulfil the ‘medium’ quality criteria of Ref. [84]. Events containing a muon candidate with a poorly measured charge-to-momentum ratio ($\sigma (q/p)\,/\,|q/p|>0.2$) are rejected. Events are required not to contain any candidate muon with large impact parameter ($|z_0|>1\,\mathrm{mm}$ or $|d_0|>0.2\,\mathrm{mm}$), as it may originate from cosmic rays. The efficiencies for electrons and muons to satisfy the reconstruction, identification, and isolation criteria are measured in samples of leptonic *Z* and $J/\psi$ decays, and corrections are applied to the simulated samples to reproduce the efficiencies in data.

The reconstruction of hadronically decaying tau leptons is based on information from tracking in the ID and three-dimensional clusters in the electromagnetic and hadronic calorimeters. The tau reconstruction algorithm is seeded by jets reconstructed as described above but with $p_{\mathrm T}>10\mathrm \,GeV$ and $|\eta |<2.5$. The reconstructed energies of the hadronically decaying tau candidates are corrected to the tau energy scale, which is calibrated based on simulation and in-situ measurements using $Z \rightarrow \tau \tau$ decays. Tau neutrinos from the tau lepton decay are not taken into account in the reconstruction and calibration of the tau energy and momentum. Hadronic tau decay candidates are required to have one or three associated charged-particle tracks (prongs) and the total electric charge of those tracks must be $\pm 1$ times the electron charge. To improve the discrimination between hadronically decaying tau leptons and jets, electrons, or muons, multivariate algorithms are used [85]. The tau identification algorithm is based on a boosted decision tree (BDT) method. The BDT algorithms use various track and cluster variables as input to discriminate tau leptons from jets. For 1-prong (3-prong) tau candidates, the signal efficiencies are 60% (50%), 55% (40%), and 45% (30%) for the ‘loose’, ‘medium’, and ‘tight’ working points, respectively. In the following, tau candidates are required to satisfy the medium identification criteria for jet discrimination (‘medium’ tau candidates), unless otherwise stated. For electron discrimination, an overlap-based veto is used for 1-prong tau candidates. This requirement has about 95% efficiency, and a rejection factor from 10 to 50 depending on the $\eta$ range. Tau candidates are required to have $p_{\text {T}} > 20\mathrm \,GeV$ and $\vert \eta \vert < 2.47$, excluding the transition region between the barrel and end-cap calorimeters ($1.37< |\eta | < 1.52$).

The simulation is corrected for differences in the efficiencies of the tau identification at both trigger and reconstruction level between data and simulation. For hadronically decaying tau leptons originating from prompt gauge boson decays, the corrections are calculated with a *tag-and-probe* method in a sample of $Z \rightarrow \tau \tau$ events where one tau lepton decays hadronically and the other leptonically into a muon and two neutrinos [86].

The measurement of the missing transverse momentum vector, $\mathbf {p}_\mathrm {T}^\mathrm {miss}$, and its magnitude, $E_{\text {T}}^{\text {miss}}$, is based on the negative vectorial sum of the $\mathbf {p_{\text {T}}}$ of all identified jets, tau candidates, electrons, photons, muons, and an additional soft term. The soft term is constructed from all high-quality tracks that are associated with the primary vertex but not with any identified particle or jet. In this way, the missing transverse momentum is adjusted for the best calibration of the jets and the other identified particles, while maintaining pile-up independence in the soft term  [87, 88].

With the reconstruction methods described above, it is possible that the same observables (tracks, calorimetric clusters) are assigned to several objects. This possible double counting of reconstructed objects is resolved in the following order. Tau candidates close to electron or muon candidates ($\Delta \mathbf {R} < 0.2$, where $\Delta \mathbf {R} = \sqrt{(\Delta y)^2 + (\Delta \phi )^2}~$) are removed, as are electrons that share a track with a muon. For electrons close to a jet ($\Delta \mathbf {R} < 0.4$), the electron is removed, except when $\Delta \mathbf {R} < 0.2$ and the jet is not *b*-tagged, in which case the jet is removed. Any remaining jet within $\Delta \mathbf {R} = 0.4$ of a muon or tau candidate is removed.

## Event selection

The events used in this analysis passed either an *asymmetric di-tau* trigger or a combined *di-tau +* $E_{\text {T}}^{\text {miss}}$ trigger. The asymmetric di-tau trigger requires the identification of two hadronically decaying tau candidates with $p_{\mathrm {T}, \tau _1}>$ 85 GeV and $p_{\mathrm {T}, \tau _2}>$ 50 GeV at trigger level for the leading and next-to-leading tau candidates respectively. Two tau candidates with $p_{\mathrm {T}, \tau _1}>$ 35 GeV and $p_{\mathrm {T}, \tau _2}>$ 25 GeV at trigger level, and $E_{\text {T}}^{\text {miss}}$ > 50 GeV (at uncalibrated electromagnetic scale) are required by the di-tau + $E_{\text {T}}^{\text {miss}}$ trigger. In events selected by the di-tau + $E_{\text {T}}^{\text {miss}}$ trigger, the reconstructed $E_{\text {T}}^{\text {miss}}$ must be larger than 150 GeV. The trigger efficiency for correctly identified tau leptons is $\sim$ 80% for events where, at reconstruction level, the leading tau candidate has $p_{\text {T}}$ > 95 (50) GeV, and the next-to-leading tau candidate has $p_{\text {T}}$ > 65 (40) GeV for the asymmetric di-tau (di-tau + $E_{\text {T}}^{\text {miss}}$) trigger.

**Table 1:** Signal region definitions

SR-lowMass	SR-highMass	
At least one opposite-sign tau pair		
*b*-jet veto		
*Z*-veto		
At least two medium tau candidates	At least one medium and one tight tau candidates	
—	$m(\tau _1, \tau _2)>$ 110 GeV	
$m_\mathrm {T2}>$ 70 GeV	$m_\mathrm {T2}>$ 90 GeV	
Di-tau+$E_{\text {T}}^{\text {miss}}$ trigger	di-tau+$E_{\text {T}}^{\text {miss}}$ trigger	Asymmetric di-tau trigger
$E_{\text {T}}^{\text {miss}}>$ 150 GeV	$E_{\text {T}}^{\text {miss}}>$ 150 GeV	$E_{\text {T}}^{\text {miss}}>$ 110 GeV
${p}_{\mathrm {T}, \tau _1}>$ 50 GeV	${p}_{\mathrm {T}, \tau _1}>$ 80 GeV	${p}_{\mathrm {T}, \tau _1}>$ 95 GeV
${p}_{\mathrm {T}, \tau _2}>$ 40 GeV	${p}_{\mathrm {T}, \tau _2}>$ 40 GeV	${p}_{\mathrm {T}, \tau _2}>$ 65 GeV

Events are required to have at least two tau candidates with opposite electric charge. The reconstructed mass of any opposite-sign (OS) tau pair must be larger than 12 GeV to remove tau leptons originating from decays of low-mass resonances. This requirement has negligible effect on the signal efficiency. Two of the reconstructed tau candidates must satisfy the $p_{\text {T}}$ requirements to be in the region where the trigger efficiency is constant (see Table 1).

To further discriminate the SUSY signal events from SM background processes, additional requirements are applied to define the signal region (SR) selections. To reject events from SM processes containing a top quark, selected events must not contain any *b*-tagged jet (*b-jet veto*). To suppress SM backgrounds with a *Z* boson, events are selected by requiring that the reconstructed mass of all oppositely charged tau pairs, $m(\tau _1, \tau _2)$, must not be within 10 GeV of the mean visible *Z* boson mass^3^ (79 GeV). This requirement is referred to as the *Z-veto*. An upper bound on the *stransverse* mass $m_\mathrm {T2}$ [89, 90] is imposed to reduce contributions from $t\bar{t}$ and *WW* events. The $m_\mathrm {T2}$variable is defined as:

$$\begin{aligned} m_\mathrm {T2}= \min _{\mathbf {q}_\mathrm {T}}\left[ \max \left( m_{{\mathrm T}, \tau _1}(\mathbf {p}_{\mathrm {T}, \tau _1},\mathbf {q}_\mathrm {T}),m_{{\mathrm T}, \tau _2}(\mathbf {p}_{\mathrm {T}, \tau _2},\mathbf {p}_\mathrm {T}^\mathrm {miss}-\mathbf {q}_\mathrm {T})\right) \right] , \end{aligned}$$

where $\mathbf {p}_{\mathrm {T}, \tau _1}$ and $\mathbf {p}_{\mathrm {T}, \tau _2}$ are the transverse momenta of the two tau candidates, and $\mathbf {q}_\mathrm {T}$ is the transverse momentum vector that minimises the larger of the two transverse masses $m_{{\mathrm T}, \tau _1}$ and $m_{{\mathrm T}, \tau _2}$. The latter masses are defined by

$$\begin{aligned} m_{{\mathrm T}}(\mathbf {p}_\mathrm {T},\mathbf {q}_\mathrm {T}) = \sqrt{2(p_{\text {T}} q_\mathrm {T}-\mathbf {p}_\mathrm {T}\cdot \mathbf {q}_\mathrm {T})}. \end{aligned}$$

In events where more than two tau candidates are selected, $m_\mathrm {T2}$ is computed among all possible tau pairs and the combination leading to the largest value is chosen. For $t\bar{t}$ and *WW* events, in which two *W* bosons decay leptonically and $\mathbf {p}_\mathrm {T}^\mathrm {miss}$ is the sum of the transverse momenta of the two neutrinos, the $m_\mathrm {T2}$ distribution has a kinematic end-point at the *W* mass. For large mass differences between the next-to-lightest neutralinos, the charginos, or the staus and the lightest neutralino, the $m_\mathrm {T2}$ distribution for signal events extends significantly beyond this end-point.

Two SRs based on large $m_\mathrm {T2}$ and $E_{\text {T}}^{\text {miss}}$ requirements are defined. SR-lowMass (SR-highMass) is designed to cover signal models where the mass difference between the  and  is smaller (larger) than 200 GeV. In SR-lowMass, only the di-tau+$E_{\text {T}}^{\text {miss}}$ trigger is used. This trigger has high efficiency in selecting events with tau leptons originating from  and  decays in models where the mass difference between the parent particle and the  is small. The main discriminating requirement is $m_\mathrm {T2}>$ 70 GeV.

In SR-highMass, events are selected by the di-tau+$E_{\text {T}}^{\text {miss}}$ trigger or by the asymmetric di-tau trigger. If the event is selected by the di-tau+$E_{\text {T}}^{\text {miss}}$ trigger, the leading tau candidate threshold is raised to ${p}_{\mathrm {T}, \tau _1}>$ 80 GeV. If the event is selected by the asymmetric di-tau trigger, $E_{\text {T}}^{\text {miss}}>$ 110 GeV is required. At least one of the tau candidates must satisfy the tight identification criteria for jet discrimination (‘tight’ tau candidate). In addition, the two leading tau candidates must satisfy $m(\tau _1, \tau _2) > 110$ GeV and $m_\mathrm {T2}>$ 90 GeV. The requirements for both SRs are summarised in Table 1. The two SRs are not mutually exclusive.

## Standard model background estimation

The main SM processes contributing to the selected final states are multi-jet, *W* + jets and diboson production. Background events may contain a combination of ‘real’ tau leptons, defined as correctly identified prompt tau leptons, or ‘fake’ tau leptons, which can originate from a misidentified light-flavour quark or gluon jet, an electron, or a muon.

In multi-jet events nearly all tau candidates are misidentified jets. The multi-jet contribution in the SRs is estimated from data, as described in Sect. 6.1. The contribution arising from heavy-flavour multi-jet events containing a real tau lepton from the heavy-flavour quark decay is included in the multi-jet estimate. The contribution of *W* + jets events, which contain one real tau lepton from the *W* decay and one or more misidentified jets, is estimated from MC simulation and normalised to data in a dedicated control region (CR), as described in Sect. 6.2.

Diboson production contributes mainly with events containing real tau leptons originating from *WW* and *ZZ* decaying into a $\tau \tau \nu \nu$ final state. Additional SM backgrounds arise from *Z* + jets production, or events that contain a top quark or a top-quark pair in association with jets or additional *W* or *Z* bosons (collectively referred to as *top* background in the following). The contribution from real tau leptons exceeds 90% in *Z* + jets and diboson production, and ranges from 45% to 75% in backgrounds containing top quarks according to the MC simulation. The contribution of fake tau leptons from heavy-flavour decays in jets is found to be negligible in MC simulation. To estimate the irreducible background, which includes diboson, *Z* + jets and top events, only MC simulated samples are used, as described in Sect. 6.3.

The sources of systematic uncertainty in the background estimates are described in Sect. 7. For each signal region a simultaneous fit based on the profile likelihood method [91] is performed to normalise the multi-jet and *W* + jets background estimates and propagate systematic uncertainties, as described in Sect. 6.4.

### Multi-jet background estimation

**Fig. 2:**
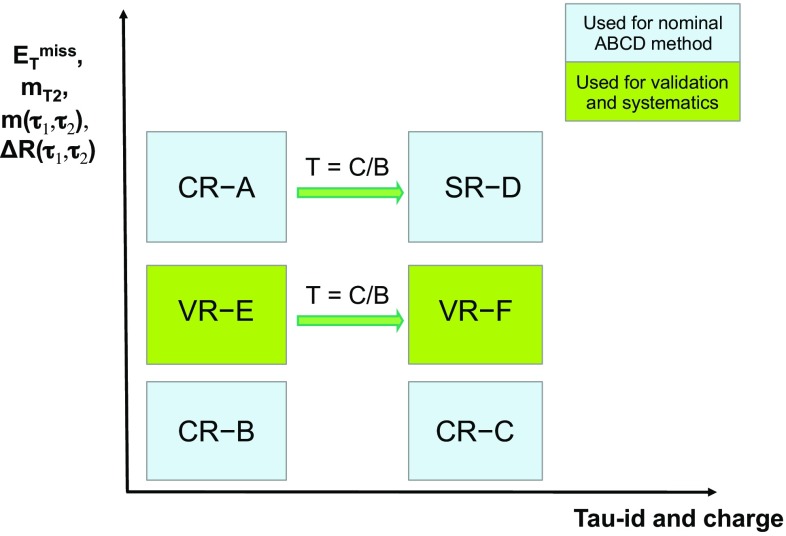
Illustration of the ABCD method for the multi-jet background determination. The control regions A, B, C, and signal region D for the ABCD method described in the text (labelled as CR-A, CR-B, CR-C and SR-D) are drawn as light blue boxes. Shown in green and labelled as VR are the regions E and F, which are used to validate the ABCD method and to estimate the systematic uncertainty. The definition of all regions used in the ABCD method can be found in Table 2

One of the dominant backgrounds in the SRs originates from jets misidentified as tau leptons in multi-jet production. It accounts for 35% (31%) of the total SM contribution in SR-highMass (SR-lowMass). This contribution is estimated from data using the so-called *ABCD* method. All regions used for the ABCD method are schematically drawn in Fig. 2. Four exclusive regions, labelled as A, B, C, and D, are defined in a two-dimensional plane as a function of two (or more) discriminating variables that are assumed to be uncorrelated. The ratio of events in the regions C and B is then equal to that in the regions D and A. The number of events in region D, $N_{\mathrm D}$, can therefore be calculated from that in region A, $N_{\mathrm A}$, multiplied by the transfer factor $\mathrm {T}=N_{\mathrm C}/N_{\mathrm B}$. The region D corresponds to one of the SRs defined in Sect. 5 (SR-lowMass or SR-highMass), whereas the regions A, B, and C are control regions defined accordingly. In the following, the regions A, B, C, D are labelled as CR-A, CR-B, CR-C, and SR-D. The definition of the regions used in the ABCD method for the multi-jet estimation is given in Table 2.

The tau identification criteria (loose, medium or tight as defined in Sect. 4), the sign of the electric charges of the two taus (OS or same sign, SS), $m(\tau _1,\tau _2)$, $\Delta R(\tau _1,\tau _2)$, $m_\mathrm {T2}$, and $E_{\text {T}}^{\text {miss}}$ are used to define CR-A, CR-B, and CR-C. Furthermore, two sets of validation regions (VR), VR-E and VR-F, are defined corresponding to each SR. The validation regions are used to verify the extrapolation of the ABCD estimation to the SRs and to estimate the systematic uncertainty from the residual correlation between the tau identification and charge requirements, and the kinematic variables $m_\mathrm {T2}$ and $E_{\text {T}}^{\text {miss}}$.

**Fig. 3:**
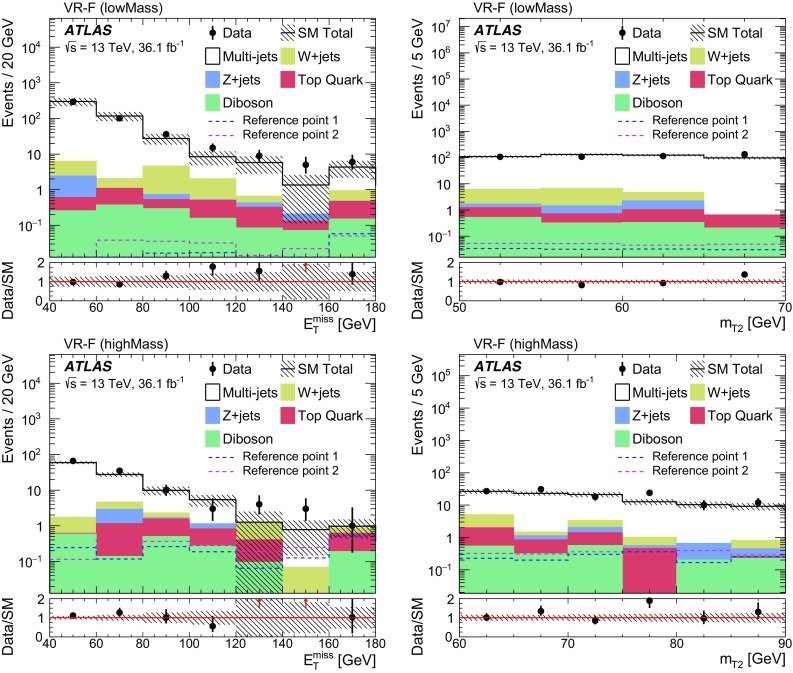
The $E_{\text {T}}^{\text {miss}}$ (left) and $m_\mathrm {T2}$ (right) distributions in the multi-jet background VR-F for SR-lowMass (top) and VR-F for SR-highMass (bottom). The stacked histograms show the contribution of the non-multi-jet SM backgrounds from MC simulation. The multi-jet contribution is estimated from data using the ABCD method. The hatched bands represent the combined statistical and systematic uncertainties in the sum of the SM backgrounds shown. For illustration, the distributions of the SUSY reference points (defined in Sect. 3) are also shown as dashed lines. The last bin in the left panels includes the overflow events

In all validation regions and both sets of CR-B and CR-C, the events passed a *di-tau* trigger instead of the di-tau + $E_{\text {T}}^{\text {miss}}$ trigger, due to the low $E_{\text {T}}^{\text {miss}}$ requirements. The di-tau trigger requires the identification of two hadronically decaying tau candidates with transverse momenta exceeding the same set of thresholds as described in Sect. 5 for the di-tau + $E_{\text {T}}^{\text {miss}}$ trigger. The di-tau trigger was prescaled during all 2016 data-taking.

**Table 2:** Definition of the regions used in the ABCD method for the multi-jet estimation in SR-lowMass (left) and SR-highMass (right). Only those requirements that are different in the CRs/VRs with respect to the SRs are listed

CR-A	SR-D (SR-lowMass)	CR-A	SR-D (SR-highMass)
Di-tau+$E_{\text {T}}^{\text {miss}}$ trigger		Di-tau+$E_{\text {T}}^{\text {miss}}$ or asymmetric di-tau trigger	
$\,\ge$ 2 loose tau leptons (SS)	$\ge$ 2 medium tau leptons (OS)	$\ge$ 2 loose tau leptons (OS)	$\ge$ 2 medium tau leptons (OS)
$\,m(\tau _1,\tau _2)<$ 250 GeV	—	< 1 medium tau < 1 tight tau leptons	$\ge$ 1 tight tau lepton
$\,\Delta R(\tau _1,\tau _2)>$ 1.5	—	$\Delta R(\tau _1,\tau _2)>$ 1.8	—
$\,E_{\text {T}}^{\text {miss}}>$ 150 GeV	$E_{\text {T}}^{\text {miss}}>$ 150 GeV	$E_{\text {T}}^{\text {miss}}>$ 110 GeV	$E_{\text {T}}^{\text {miss}}>$ 110 GeV
$\,m_\mathrm {T2}>$ 70 GeV	$m_\mathrm {T2}>$ 70 GeV	$m_\mathrm {T2}>$ 90 GeV	$m_\mathrm {T2}>$ 90 GeV

VR-E	VR-F	VR-E	VR-F
Di-tau trigger		Di-tau or asymmetric di-tau trigger	
$\,\ge$ 2 loose tau leptons (SS)	$\ge$ 2 medium tau leptons (OS)	$\ge$ 2 loose tau leptons (OS)	$\ge$ 2 medium tau leptons (OS)
$\,m(\tau _1,\tau _2)<$ 250 GeV	—	< 1 medium tau < 1 tight tau leptons	$\ge$ 1 tight tau lepton
$\,\Delta R(\tau _1,\tau _2)>$ 1.5	—	$\Delta R(\tau _1,\tau _2)>$ 1.8	—
$\,E_{\text {T}}^{\text {miss}}>$ 40 GeV	$E_{\text {T}}^{\text {miss}}>$ 40 GeV	$E_{\text {T}}^{\text {miss}}>$ 40 GeV	$E_{\text {T}}^{\text {miss}}>$ 40 GeV
50 $<m_\mathrm {T2}<$ 70 GeV	50 $<m_\mathrm {T2}<$ 70 GeV	60 $<m_\mathrm {T2}<$ 90 GeV	60 $<m_\mathrm {T2}<$ 90 GeV

CR-B	CR-C	CR-B	CR-C
Di-tau trigger		Di-tau or asymmetric di-tau trigger	
$\,\ge$ 2 loose tau leptons (SS)	$\ge$ 2 medium tau leptons (OS)	$\ge$ 2 loose tau leptons (OS)	$\ge$ 2 medium tau leptons (OS)
$\,m(\tau _1,\tau _2)<$ 250 GeV	—	< 1 medium tau < 1 tight tau leptons	$\ge$ 1 tight tau
$\,\Delta R(\tau _1,\tau _2)>$ 1.5	—	$\Delta R(\tau _1,\tau _2)>$ 1.8	—
$\,E_{\text {T}}^{\text {miss}}>$ 40 GeV	$E_{\text {T}}^{\text {miss}}>$ 40 GeV	$E_{\text {T}}^{\text {miss}}>$ 40 GeV	$E_{\text {T}}^{\text {miss}}>$ 40 GeV
20 $<m_\mathrm {T2}<$ 50 GeV	20 $<m_\mathrm {T2}<$ 50 GeV	10 $<m_\mathrm {T2}<$ 60 GeV	10 $<m_\mathrm {T2}<$ 60 GeV

The number of multi-jet events in the control and validation regions is estimated from data after subtraction of other SM contributions estimated from MC simulation. In both CR-B and VR-E, more than 86% of the events come from multi-jet production, whereas for CR-A and CR-C the multi-jet purity is larger than 47 and 68%, respectively. In VR-F the multi-jet purity is larger than 90%. Agreement between data and the estimated SM background is found for the $E_{\text {T}}^{\text {miss}}$ and $m_\mathrm {T2}$ distributions in the validation regions, as shown in Fig. 3. The correlation between the tau identification and charge and the kinematic variables is checked by studying the variation of the transfer factor $\mathrm {T}$ as a function of the kinematic variables $m_\mathrm {T2}$ and $E_{\text {T}}^{\text {miss}}$, and is found to be negligible. The results of the ABCD method are summarised in Table 3.

The signal contamination in a certain region is defined as the ratio of the number of signal events to the sum of the number of signal events and SM background processes. The signal contamination in CR-A for both SRs ranges from a few percent to 30–50% for a few signal models, and it is taken into account in the simultaneous fit described in Sect. 6.4. The largest contaminations are found for a  mass of 400 GeV and massless  for  production, and for a  mass of 300 GeV and massless  for  production. The possible presence of non-SM event contamination in CR-A was tested and proved not to change the fit results significantly.

**Table 3:** The MC predicted backgrounds in the multi-jet control regions, including the statistical uncertainties, and the expected multi-jet contribution (in italics), obtained by subtracting the MC contributions from observed data (in bold). Predicted event yields for the SUSY reference points (defined in Sect. 3) in the control regions are also shown. The estimated multi-jet contribution in the SRs is given in the last column including both the statistical and systematic uncertainties. The details of the systematic uncertainties reported here are discussed in Sect. 7

SR	Sample	CR-B	CR-C	CR-A	$\mathrm {T}$ = C/B	Multi-jet in SR-D
lowMass	$\mathbf {Data }$	$\mathbf { 556 }$	$\mathbf { 674 }$	$\mathbf { 8 }$		
	*Z* + jets	3.4 ± 2.1	19 ± 5	0.8 ± 0.4		
	*W* + jets	8.9 ± 1.8	20 ± 5	1.8 ± 1.0		
	Diboson	0.94 ± 0.12	3.3 ± 0.2	0.29 ± 0.07	1.16	4.3
	Top	1.61 ± 0.30	4.7 ± 0.5	1.4 ± 1.1	± 0.07	± 4.0
	*Multi-jet*	$\textit{541 }\pm \textit{ 24}$	$\textit{627 }\pm \textit{ 27}$	$\textit{3.7 }\pm \textit{ 1.6}$		
	Reference point 1	0.06 ± 0.01	0.16 ± 0.02	1.68 ± 0.16		
highMass	$\mathbf {Data }$	$\mathbf { 1565 }$	$\mathbf { 836 }$	$\mathbf { 5 }$		
	*Z* + jets	56 ± 31	93 ± 42	0.02 ± 0.29		
	*W* + jets	151 ± 22	125 ± 17	1.1 ± 0.4		
	Diboson	9.6 ± 1.1	20.5 ± 2.0	0.8 ± 0.4	0.43	1.3
	Top	9.2 ± 1.5	25.4 ± 3.4	0.01 ± 0.01	± 0.04	± 1.1
	*Multi-jet*	$\textit{1340 }\pm \textit{50}$	$\textit{ 570}\pm \textit{ 50 }$	$\textit{3.1} \pm \textit{0.6}$		
	Reference point 2	0.53 ± 0.08	2.37 ± 0.21	1.92 ± 0.16		

### *W* + jets background estimation

The production of *W* + jets events with at least one misidentified tau lepton is an important background, accounting for about 13% (20%) of the expected SM background in SR-lowMass (SR-highMass). A dedicated control region (*W*-CR) is used to normalise the *W* + jets MC estimate to data. To suppress multi-jet contamination, the *W*-CR is enriched in events where the *W* boson decays leptonically into a muon and a neutrino. Events are selected with a single-muon trigger, using the lowest unprescaled $p_{\text {T}}$ thresholds available. Events containing exactly one isolated muon and one candidate tau lepton with opposite electric charge are selected. The muon is required to have $p_{\text {T}}$  > 40 GeV. In addition, the muon must satisfy the ‘GradientLoose’ [84] isolation requirements, which rely on the use of track and calorimeter based variables and implement a set of $\eta$- and $p_{\text {T}}$-dependent criteria. Compatibility of the signal lepton tracks with the primary vertex is enforced by requiring $|z_0\sin \theta | < 0.5~\mathrm {mm}$, where $z_0$ is the longitudinal impact parameter. In addition, the transverse impact parameter, $d_0$, divided by its uncertainty, $\sigma (d_0)$, must satisfy $|d_0/\sigma (d_0)|<3$ for the muon. The tau candidate must satisfy the medium tau identification criteria and is required to have $p_{\text {T}}$ > 50 GeV.

The contribution from events with top quarks is suppressed by rejecting events containing *b*-tagged jets. To reduce the contribution from *Z* + jets production, the transverse mass of the $\mu +E_{\text {T}}^{\text {miss}}$ system, $m_{\mathrm {T},\mu }>$ 50 GeV, the sum of the transverse mass of the $\tau +E_{\text {T}}^{\text {miss}}$ and $\mu +E_{\text {T}}^{\text {miss}}$ systems, $m_{{\mathrm T},\tau } + m_{{\mathrm T},\mu }>$ 80 GeV, and the angular separation between the muon and the tau lepton $\Delta R(\mu ,\tau )>$ 0.5 are required. To further suppress diboson and top-quark contributions, $m_{\mathrm {T},\mu }<$ 150 GeV is required. To be close to the SR definition, $E_{\text {T}}^{\text {miss}}>$ 60 GeV and the invariant mass of the muon and tau lepton, $m(\mu , \tau )>$ 70 GeV are required. Events in the *W*-CR are selected by requiring low $m_\mathrm {T2}$, while a high $m_\mathrm {T2}$ region is used to validate the *W* + jets estimate (*W* validation region, *W*-VR). The definitions of the *W*-CR and *W*-VR are given in Table 4.

The multi-jet contribution in the *W*-CR (*W*-VR) is estimated using the so-called *OS–SS* method by counting the number of events in data satisfying the same requirements as the *W*-CR (*W*-VR) but with the electric charge of the two leptons having the same sign (SS). Events from SM processes other than multi-jet production are subtracted from the data counts in the SS region using MC simulation. The OS–SS method relies on the fact that in the multi-jet background the ratio of SS to OS events is close to unity, while a significant difference from unity is expected for *W* + jets production. The latter is dominated by *gu* / *gd*-initiated processes that often give rise to a jet originating from a quark, the charge of which is anti-correlated with the *W* boson charge. Based on studies with simulated samples, a systematic uncertainty of 100% is assigned to the multi-jet estimate in the *W*-CR.

**Fig. 4:**
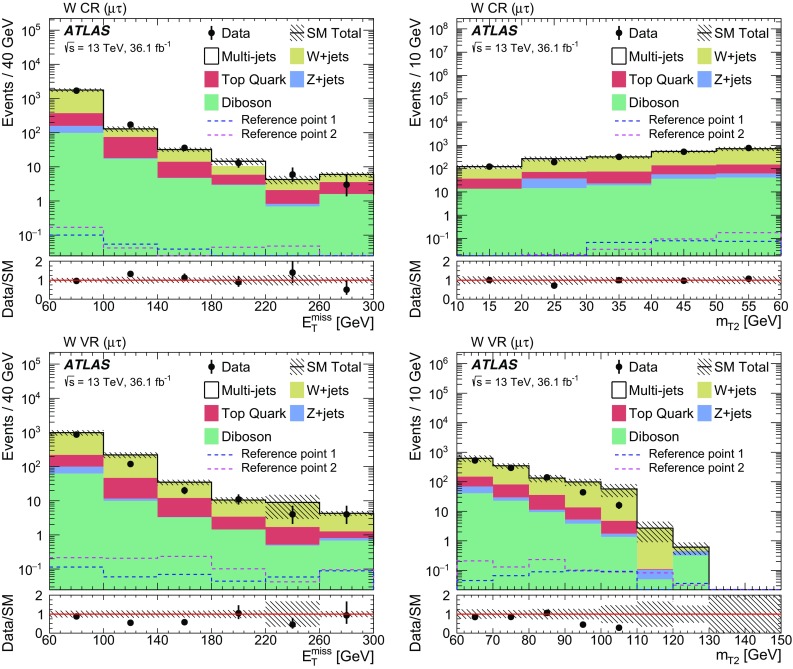
The $E_{\text {T}}^{\text {miss}}$ (left) and $m_\mathrm {T2}$(right) distributions in the *W*-CR (top) and *W*-VR (bottom) regions. The SM backgrounds other than multi-jet production are estimated from MC simulation. The contribution of *W* + jets events is scaled to the fit result. The multi-jet contribution is estimated from data using the *OS–SS* method. The hatched bands represent the combined statistical and systematic uncertainties of the total SM background. For illustration, the distributions of the SUSY reference points defined in Sect. 3 are also shown as dashed lines. The lower panels show the ratio of data to the SM background estimate. The last bin includes the overflow events

The event yields in the *W*-CR and *W*-VR are given in Table 5. The purity of the selection in *W* + jets events is around 72% (77%) in the *W*-CR (*W*-VR). Agreement between data and SM predictions is observed. The signal contamination in the *W*-CR and *W*-VR is negligible. Distributions of the kinematic variables defining the SRs are shown in Fig. 4, in which the contribution of *W* + jets events is scaled with the normalisation factor 1.02 obtained from the fit described in Sect. 6.4. The discrepancy between observed data and predictions at $m_\mathrm {T2}>$ 90 GeV in the *W*-VR is due to events with different kinematics from the SRs, with either $E_{\text {T}}^{\text {miss}}<$ 150 GeV or where the muon has $p_{\text {T}}$  < 60 GeV.

**Table 4:** The *W*-CR (left) and *W*-VR (right) definitions

*W*-CR	*W*-VR
One isolated muon and one medium tau lepton with opposite sign	
*b*-jet veto	
$m(\mu , \tau )>$ 70 GeV	
$E_{\text {T}}^{\text {miss}}>$ 60 GeV	
50 $< m_{\mathrm {T},\mu }<$ 150 GeV	
$m_{\mathrm {T},\mu }+m_{\mathrm {T},\tau }>$ 80 GeV	
0.5 $< \Delta R(\mu ,\tau )<$ 3.5	0.5 $< \Delta R(\mu ,\tau )<$ 4.5
10 $< m_\mathrm {T2}<$ 60 GeV	$m_\mathrm {T2}>$ 60 GeV

**Table 5:** Event yields in the *W*-CR and *W*-VR. The SM backgrounds other than multi-jet production are estimated from MC simulation. The contribution of *W* + jets events is scaled with the normalisation factor obtained from the fit. The multi-jet contribution is estimated from data using the OS–SS method. In the *W*-VR the multi-jet estimation with the OS-SS method yields a negative contribution, which is set to zero. Predicted event yields for the SUSY reference points (defined in Sect. 3) are also shown. The uncertainties given are the sum in quadrature of statistical and systematic uncertainties. The correlation of systematic uncertainties among control and validation regions and background processes is fully taken into account in the fit

Sample	*W*-CR	*W*-VR
Data	1928	1023
SM total	$1930 \pm 50$	$1260 \pm 440$
$W+$jets	$1395 \pm 130$	$980 \pm 410$
$Z+$jets	$60 \pm 28$	$39 \pm 15$
Diboson	$125 \pm 24$	$78 \pm 20$
Top quark	$290 \pm 80$	$170 \pm 60$
Multi-jet	$60 \pm 60$	$0 \pm 100$
Reference point 1	$0.22 \pm 0.07$	$0.44 \pm 0.08$
Reference point 2	$0.33 \pm 0.08$	$0.87 \pm 0.11$

### Irreducible background estimation

Irreducible SM backgrounds arise mainly from $t\bar{t}$, single top quark, $t\bar{t}$+*V*, *Z* + jets, and diboson (*WW*, *WZ* and *ZZ*) processes and are estimated with MC simulation. Other SM backgrounds are negligible.

The inclusive contribution from $t\bar{t}$, single top quark, $t\bar{t}$+*V* and *Z* + jets amounts to about 18% (13%) of the total background in SR-highMass (SR-lowMass). The MC estimates are validated in regions enriched in *Z* + jets and top-quark events. For both regions, the events passed either the combined di-tau + $E_{\text {T}}^{\text {miss}}$ trigger or the asymmetric di-tau trigger. Events are required to have at least two tau candidates with opposite electric charge, $E_{\text {T}}^{\text {miss}}$  > 150 GeV, and leading (sub-leading) tau $p_{\mathrm T}>$ 50 (40) GeV. In the *Z* + jets validation region (*Z*-VR), at least two tau candidates must satisfy the medium tau identification criteria. To suppress top-quark backgrounds, events containing *b*-tagged jets are vetoed. To further enhance the purity of *Z* + jets events, $m_\mathrm {T2}$ < 10 GeV is required. In the top-quark validation region (Top-VR), at least one tau candidate must satisfy the medium tau identification criteria. To increase the contribution from top-quark events, events must contain at least one *b*-tagged jet with $p_{\mathrm T} > 20$ GeV and must be kinematically compatible with $t\bar{t}$ production (top-tagged) through the use of the *contransverse* mass $m_{\mathrm {CT}}$ [92]. The scalar sum of the $p_{\mathrm T}$ of the two tau leptons and of at least one combination of two jets in an event must exceed 100 GeV. Top-tagged events are required to possess $m_{\mathrm {CT}}$ values calculated from combinations of jets and tau leptons consistent with the expected bounds from $t\bar{t}$ events as described in Ref. [93]. The *Z*-VR and Top-VR requirements are summarised in Table 6.

The diboson background accounts for 26% (43%) of the total SM contribution in the SR-highMass (SR-lowMass) and mainly arises from $WW\rightarrow \tau \nu \tau \nu$ and $ZZ \rightarrow \tau \tau \nu \nu$ events, in which more than 96% of the contribution is from events with two real tau leptons according to the MC simulation. To validate the MC modelling and normalisation of the *WW* (*ZZ*) process, a validation region *WW*-VR (*ZZ*-VR) with an enriched $WW \rightarrow e \nu \mu \nu$ ($ZZ \rightarrow e e \nu \nu$ or $ZZ \rightarrow \mu \mu \nu \nu$) contribution is defined. For *WW*-VR, events with two isolated leptons ($\ell = e$ or $\mu$) with different flavour and opposite sign are selected, while for *ZZ*-VR, events with two isolated leptons with same flavour and opposite sign are selected. To keep the phase space similar to the SRs, *WW*-VR (*ZZ*-VR) is defined to be close to the SRs except for the selected objects being a light-lepton pair. Top-tagged events are vetoed to suppress the $t\bar{t}$ contribution in *WW*-VR. To suppress the *Z* + jets contribution in *ZZ*-VR, $\Delta R(\ell ,\ell )<$ 1.5 is applied; the requirement $|m_{\ell \ell }-m_Z|<$ 15 GeV is used to enrich the *ZZ* contribution. The definitions of *WW*-VR and *ZZ*-VR are summarised in Table 7.

**Fig. 5:**
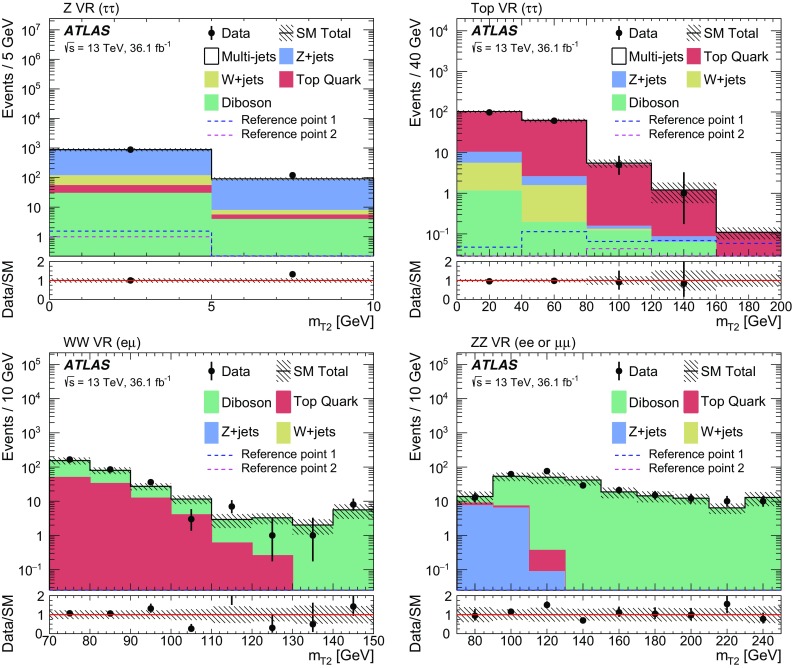
The $m_\mathrm {T2}$ distribution in the *Z*-VR (top left), Top-VR (top right), *WW*-VR (bottom left) and *ZZ*-VR (bottom right) regions. The SM backgrounds other than multi-jet production are estimated from MC simulation. The multi-jet contribution is negligible and not considered in *WW*-VR and *ZZ*-VR, while in *Z*-VR and Top-VR it is estimated from data using the ABCD method, using CRs obtained with the same technique used for the SRs, and described in Sect. 6.1. The hatched bands represent the combined statistical and systematic uncertainties of the total SM background. For illustration, the distributions of the SUSY reference points (defined in Sect. 3) are also shown as dashed lines. The lower panels show the ratio of data to the SM background estimate. The last bin includes the overflow events except for the upper left panel

The purity of the selection in *Z* + jets and $t\bar{t}$ events is above 80% in the respective validation regions, and the purity of the selection in *WW* (*ZZ*) events is around 65% (92%) in *WW*-VR (*ZZ*-VR). Agreement between data and the SM prediction is observed in all validation regions. The $m_\mathrm {T2}$distributions in the *Z*-VR, Top-VR, *WW*-VR and *ZZ*-VR are shown in Fig. 5.

### Statistical analysis

The statistical interpretation of the results is performed using the profile likelihood method implemented in the HistFitter framework [94]. Three types of fits are performed for each SR.

**Table 6:** The *Z*-VR (left) and Top-VR (right) definitions

*Z*-VR	Top-VR
At least one opposite-sign tau lepton pair	
Tau $p_{\text {T}}$ > 50, 40 GeV	
$E_{\text {T}}^{\text {miss}}>$ 60 GeV	
At least two medium tau leptons	At least one medium and one loose tau lepton
*b*-jet veto	At least one *b*-jet
$m_\mathrm {T2}<$ 10 GeV	$m_\mathrm {T2}>$ 10 GeV
—	$m_{\mathrm {CT}}$ top-tagged

**Table 7:** The *WW*-VR (left) and *ZZ*-VR (right) definitions

*WW*-VR	*ZZ*-VR
One opposite-sign lepton (*e* or $\mu$) pair	
$\mu$ $p_{\text {T}}$ >30 GeV, *e* $p_{\text {T}}$ > 40 GeV	
Jet veto	
$m_{\ell \ell }~>$ 50 GeV	
$E_{\text {T}}^{\text {miss}}>$ 50 GeV	
$m_{\mathrm {T}, \mu }>$ 100 GeV	
$m_\mathrm {T2}>$ 70 GeV	
Two isolated leptons (*e* or $\mu$) with different flavour	Two isolated leptons (*e* or $\mu$) with same flavour
$m_{\mathrm {CT}}$ top tag veto	$\Delta R(\ell ,\ell )<$ 1.5
—	$|m_{\ell \ell }-m_Z|$ < 15 GeV

- The *background-only* fit uses as input the number of observed events in the multi-jet CR-A and *W*-CR, the expected SM contributions other than multi-jet to the multi-jet CR-A and *W*-CR, and the transfer factors, which are used to extrapolate the background of multi-jet or *W* + jets events in their control regions to these predicted in the signal regions. The free parameters in the fit are the normalisations of the *W* + jets and multi-jet contributions. The signal is assumed to be absent in this fit.
- A *model-independent limit* fit combines the data event yield in a given SR with the SM background estimate and its uncertainties obtained by the background-only fit to test whether any non-SM signal contributes to the SR. The significance of a possible excess of observed events over the SM prediction is quantified by the one-sided probability, $p (\text {signal} = 0)$ denoted by $p_0$, of the background alone to fluctuate to the observed number of events or higher using the asymptotic formula described in Ref. [91]. The presence of a non-SM signal would manifest itself in a small $p_0$ value.
- In the *model-dependent limit* fit the SUSY signal is allowed to populate both the signal and the control regions, and it is scaled by a freely floating signal normalisation factor. The background normalisation factors are also determined simultaneously in the fit. A SUSY model with a specific set of sparticle masses is rejected if the upper limit at 95% confidence level (CL) of the signal normalisation factor obtained in this fit is smaller than unity.

The likelihood function is a product of the probability density functions, one for each region contributing to the fit. The number of events in a given CR or SR is described using a Poisson distribution, the mean of which is the sum of the expected contributions from all background and signal sources. The systematic uncertainties in the expected event yields are included as nuisance parameters and are assumed to follow a Gaussian distribution with a width determined from the size of the uncertainty. Correlations between control and signal regions, and background processes are taken into account with common nuisance parameters. The fit parameters are determined by maximising the product of the Poisson probability functions and the constraints for the nuisance parameters.

## Systematic uncertainties

Systematic uncertainties have an impact on the estimates of the background and signal event yields in the control and signal regions. Uncertainties arising from experimental effects and theoretical sources are estimated.

The main sources of experimental systematic uncertainty in the SM background estimates include tau lepton and jet energy calibrations and resolution, tau lepton identification, pile-up, and uncertainties related to the modelling of $E_{\text {T}}^{\text {miss}}$ in the simulation. The uncertainties in the energy and momentum scale of each of the objects entering the $E_{\text {T}}^{\text {miss}}$ calculation are estimated, as well as the uncertainties in the soft-term resolution and scale. A variation in the pile-up reweighting of the MC simulated event samples is included to cover the uncertainty in the ratio of the predicted and measured inelastic cross section in the fiducial volume defined by $M_X>$ 13 GeV where $M_X$ is the mass of the hadronic system [95]. The main contributions to experimental systematic uncertainties in the SR-lowMass (SR-highMass) are from the tau lepton identification and energy scale around 6% (8%), jet energy scale and resolution around 11% (4%), $E_{\text {T}}^{\text {miss}}$ soft-term resolution and scale around 2% (6%), and pile-up around 8% (8%). Other contributions are less than 3%.

Theoretical uncertainties affecting the MC event generator predictions are estimated by varying the renormalisation, factorisation, and resummation scales, and the matching scale between the matrix elements and the parton shower. For *W* + jets and diboson processes, the uncertainties related to the choice of the QCD renormalisation and factorisation scales are estimated from the comparison of the nominal samples with samples with these scales varied up and down by a factor of two. Uncertainties in the resummation scale and the matching scale between the matrix elements and parton shower are evaluated by varying up and down the corresponding parameters in Sherpa by a factor of two. For *W* + jets events, the uncertainty due to the jet $p_{\text {T}}$ threshold used for parton–jet matching is estimated by comparing the baseline samples with jet $p_{\text {T}}$ threshold set to 20 GeV to samples with a threshold of 15 or 30 GeV. Sherpa is compared with MadGraph to estimate the uncertainty related to the generator choice for *W* + jets production. The total theoretical uncertainty for diboson processes in the SRs is around 15%, mainly coming from the choice of QCD renormalisation scale (4–9%) and resummation scale (around 10%). The theory uncertainty in *W* + jets production is 13–20%, and the main source is the event generator uncertainty (4–17%) and the QCD renormalisation scale (9–10%). An overall systematic uncertainty of 6% in the inclusive cross section is assigned to the diboson process. Based on previous studies [29], a total theoretical uncertainty of 25% is assigned for the top-quark and *Z* + jets contributions to the SRs.

The following sources of uncertainty are considered for the ABCD method used to determine the multi-jet background: the correlation between the tau-id, the charge requirement, and the kinematic variables $m_\mathrm {T2}$, the limited number of events in the CRs, and the subtraction of other SM backgrounds. The systematic uncertainty in the correlation is estimated by comparing the transfer factor from CR-B to CR-C to that of VR-E to VR-F. The systematic uncertainty in the non-multi-jet background subtraction in the control regions is estimated by considering the systematic uncertainty of the MC estimates of the non-multi-jet background in the CRs. Both uncertainties are of the order of 10%. The systematic uncertainty in the signal region due to the limited number of events in the control regions is estimated by taking the statistical uncertainty of the event yields in these control regions. It corresponds to the largest source of uncertainty for the ABCD method, and it reaches 21–42% for CR-A.

The systematic uncertainties on the background estimates in the SRs are summarised in Table 8. The dominant uncertainties are the multi-jet background normalisation (around 32% in both SR-lowMass and SR-highMass), and the statistical uncertainty of the MC predictions (around 18% in SR-lowMass and 24% in SR-highMass respectively).

The total uncertainty in the signal yields for the SUSY reference points defined in Sect. 3 is about 20%. The main sources of experimental uncertainty are the tau lepton identification and energy scale, jet energy scale and resolution, $E_{\text {T}}^{\text {miss}}$ soft-term resolution and scale, and pile-up: they amount to a total of about 15%. The cross-section uncertainty is taken into account as main source of theoretical uncertainty, and it varies from 3 to 20% for the considered SUSY models. SUSY models with higher chargino mass have larger uncertainties.

**Table 8:** The relative systematic uncertainty (%) in the background estimate in the SR-lowMass and SR-highMass from the leading sources. Uncertainties from different sources may be correlated, and do not necessarily add in quadrature to the total uncertainty

Source of systematic uncertainty	SR-lowMass	SR-highMass
Normalisation uncertainties of the multi-jet background	32	32
Statistical uncertainty of MC samples	18	24
Multi-jet estimation	14	13
Pile-up reweighting	8	8
Jet energy scale and resolution	11	4
Tau identification and energy scale	6	8
$E_{\text {T}}^{\text {miss}}$ soft-term resolution and scale	2	6
Total	40	38

## Results

**Fig. 6:**
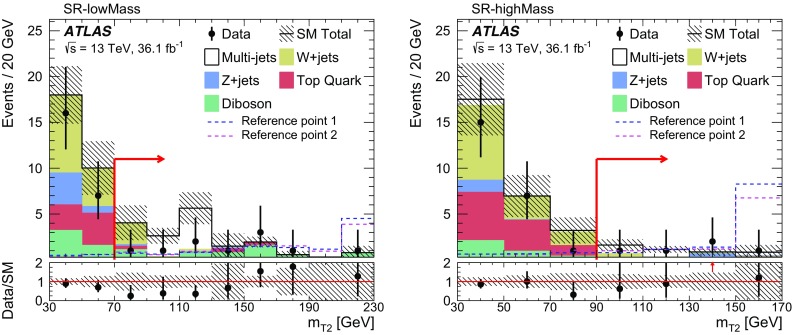
The $m_\mathrm {T2}$ distribution before the $m_\mathrm {T2}$ requirement is applied for SR-lowMass (left) and SR-highMass (right) regions, where the arrow indicates the position of the cut in the signal region. The stacked histograms show the expected SM backgrounds. The multi-jet contribution is estimated from data using the ABCD method. The contributions of multi-jet and *W* + jets events are scaled with the corresponding normalisation factors. The hatched bands represent the sum in quadrature of systematic and statistical uncertainties of the total SM background. For illustration, the distributions of the SUSY reference points (defined in Sect. 3) are also shown as dashed lines. The lower panels show the ratio of data to the total SM background estimate. The last bin includes the overflow events

The observed number of events in each signal region and the expected contributions from SM processes are given in Table 9. The contributions of multi-jet and *W* + jets events are scaled with the normalisation factors obtained from the background-only fit described in Sect. 6.4. The multi-jet normalisation with respect to the prediction from the ABCD method in the SR-lowMass (SR-highMass) is compatible with unity and has an uncertainty of around 100% (86%), due to the small number of observed events in the multi-jet CR-A. The *W* + jets normalisation is 1.02 ± 0.15. The $m_\mathrm {T2}$ distribution is shown in Fig. 6 for data, expected SM backgrounds, and the SUSY reference points defined in Sect. 3. In both signal regions, observations and background predictions are found to be compatible within uncertainties.

Upper limits at 95% CL on the number of non-SM events in the SRs are derived from the model-independent fit. All limits are calculated using the CL$_\mathrm {s}$ prescription [96]. Normalising these by the integrated luminosity of the data sample, they can be interpreted as upper limits on the visible non-SM cross section, $\sigma _{\mathrm {vis}}^{95}$, which is defined as the product of acceptance, reconstruction efficiency and production cross section. The accuracy of the limits obtained from the asymptotic formula was tested for all SRs by randomly generating a large number of pseudo-datasets and repeating the fit. Good agreement was found.

**Table 9:** Observed and expected numbers of events in the signal regions. The contributions of multi-jet and *W* + jets events are scaled with the normalisation factors obtained from the background-only fit described in Sect. 6.4. Expected event yields for the SUSY reference points (defined in Sect. 3) are also shown. The uncertainties correspond to the sum in quadrature of statistical and systematic uncertainties. The correlation of systematic uncertainties among control regions and among background processes is fully taken into account. The one-sided $p_0$-values, the observed and expected 95% CL upper limits on the visible non-SM cross section ($\sigma _{\mathrm {vis}}^{95}$) are given. Values of $p_0>0.5$ are truncated to $p_0=0.5$

SM process	SR-lowMass	SR-highMass
Diboson	5.9 ± 2.2	1.0 ± 0.8
*W* + jets	1.8 ± 1.1	0.7 ± 0.5
Top quark	1.2 ± 1.0	$0.03_{-0.03}^{+0.26}$
*Z* + jets	$0.6_{-0.6}^{+0.7}$	0.6 ± 0.5
Multi-jet	4.3 ± 4.0	1.3 ± 1.1
SM total	14 ± 6	3.7 ± 1.4
Observed	10	5
Reference point 1	$11.8 \pm 2.8$	$11.6 \pm 2.6$
Reference point 2	$11.4 \pm 2.6$	$10.0 \pm 2.1$
$p_0$	0.5	0.3
Expected $$\sigma _\text {vis}^{95}$$ [fb]	$$ { 0.31 }^{ +0.12 }_{ -0.08 }$$	$$ { 0.17 }^{ +0.08 }_{ -0.05 }$$
Observed $\sigma _\text {vis}^{95}$ [fb]	0.26	0.20

## Interpretation

**Fig. 7:**
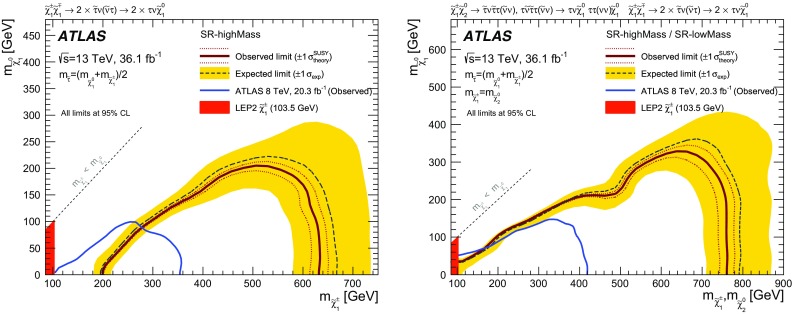
The 95% CL exclusion contours for simplified models with  production (left) and production of  and  (right). The text provides details of exclusion curves and uncertainty bands. The LEP limit on the chargino mass is also shown. Results are compared with the observed limits obtained by previous ATLAS searches [29] as blue contours

In the absence of a significant excess over the expected SM background, the observed and expected numbers of events in the signal regions are used to place exclusion limits at 95% CL using the model-dependent limit fit described in Sect. 6.4. SR-highMass is used to derive limits on  production and the best limit expected for SR-highMass and SR-lowMass is used to derive limits for the production of  and . The exclusion limits for simplified models with $x=0.5$, described in Sect. 3, are shown in Fig. 7. Only  production is assumed for the left plot, whereas both production processes are considered simultaneously for the right plot. The solid (dashed) lines show the observed (expected) exclusion contours. The band around the expected limit shows the $\pm \,1\sigma$ variations, including all uncertainties except theoretical uncertainties in the signal cross section. The dotted lines around the observed limit indicate the sensitivity to $\pm \,1\sigma$ variations of the theoretical uncertainties in the signal cross section.

Chargino masses up to 630 GeV are excluded for a massless lightest neutralino in the scenario of direct production of chargino pairs. In the case of production of chargino pairs and mass-degenerate charginos and next-to-lightest neutralinos, chargino masses up to 760 GeV are excluded for a massless lightest neutralino. Both limits apply to scenarios where the neutralinos and charginos decay solely via intermediate staus and tau sneutrinos, and with the parameter *x* equal to 0.5. These limits significantly extend previous results [29, 30] in the high chargino mass region.

**Fig. 8:**
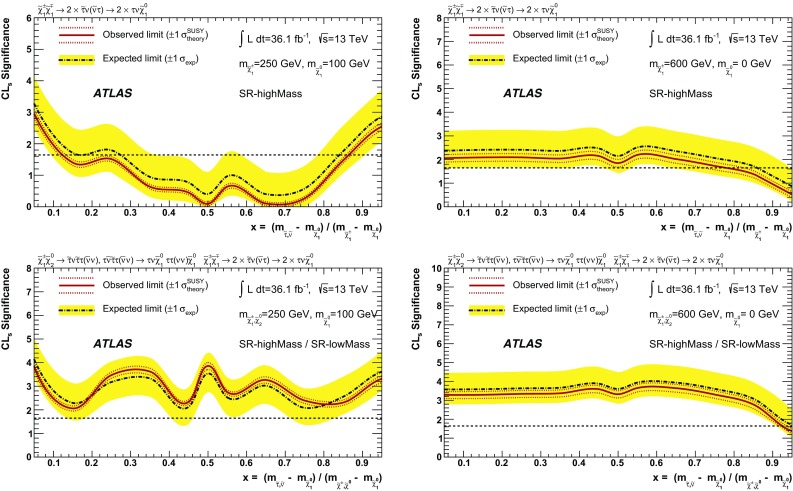
The CL$_\mathrm {s}$ significance for the benchmark models described in Sect. 3 as a function of the parameter *x*. The benchmark scenario with large mass splitting ( = 600 GeV and  = 0 GeV) is shown on the right, and the compressed benchmark scenario ( = 250 GeV and  = 100 GeV) on the left, for  production (top), and  and  production (bottom). SR-highMass is used for  production, while the SR with the best expected CL$_\mathrm {s}$ value for each point of the parameter space is used for  and  production

The impact of *x* different from 0.5 is studied by varying it between 0.05 and 0.95 for two benchmark scenarios. The CL$_\mathrm {s}$ significance as a function of the parameter *x* is shown in Fig. 8. When only  production is considered, the benchmark scenario with large mass-splitting ( = 600 GeV and massless ) can be excluded for *x* up to 0.75. For larger values of *x* the $p_{\text {T}}$ spectra of the tau leptons from the chargino decay become very soft. The compressed benchmark scenario ( = 250 GeV and  = 100 GeV) can only be excluded for the extreme cases with $x=0.05$ or $x=0.95$, since the $m_\mathrm {T2}$ requirement is more effective for models with large mass-splittings between the charginos or the staus and the lightest neutralino. Models with low values of *x* typically predict dark-matter relic density consistent with cosmological observations. For combined production of  and  the same general features are observed, but due to the higher signal yields with respect to  production alone, both benchmark scenarios can be excluded for all considered values of *x*.

## Conclusion

Searches for the electroweak production of supersymmetric particles in events with at least two hadronically decaying tau leptons are performed using 36.1 fb$^{-1}$of *pp* collision data at $\sqrt{s}=13$ TeV recorded with the ATLAS experiment at the Large Hadron Collider. Agreement between data and SM predictions is observed in two optimised signal regions. The results are used to set limits on the visible cross section for events beyond the Standard Model in each signal region. Observed upper limits on the simplified model cross-sections have been calculated and are available in [97].

Exclusion limits are placed on parameters of simplified electroweak supersymmetry models in scenarios where the neutralinos and charginos decay solely via intermediate left-handed staus and tau sneutrinos, and the mass of the $\tilde{\tau }_{\mathrm L}$ state is set to be halfway between the masses of the  and the  ($x=0.5$). Chargino masses up to 630 GeV are excluded for a massless lightest neutralino in the scenario of direct production of chargino pairs, with each chargino decaying into the lightest neutralino via an intermediate on-shell stau or tau sneutrino. An additional benchmark scenario with large mass-splitting ( = 600 GeV and massless ) can be excluded for *x* up to 0.75, whereas a compressed benchmark scenario ( = 250 GeV and  = 100 GeV) can only be excluded for the extreme cases with $x=0.05$ or $x=0.95$. In the case of production of chargino pairs and mass-degenerate charginos and next-to-lightest neutralinos, common  and  masses up to 760 GeV are excluded for a massless lightest neutralino. The additional benchmark scenarios with small and large mass-splitting can be both excluded for all considered values of *x*.

## Footnotes

1 For the interval  GeV, the chargino mass limit set by LEP degrades to $91.9 \mathrm \,GeV$.

2 ATLAS uses a right-handed coordinate system with its origin at the nominal interaction point (IP) in the centre of the detector, and the *z*-axis along the beam line. The *x*-axis points from the IP to the centre of the LHC ring, and the *y*-axis points upwards. Cylindrical coordinates $(r,\phi )$ are used in the transverse plane, $\phi$ being the azimuthal angle around the *z*-axis. Observables labelled *transverse* refer to the projection into the *x*–*y* plane. The pseudorapidity is defined in terms of the polar angle $\theta$ by $\eta =-\ln \tan (\theta /2)$.

3 The mean visible *Z* boson mass is defined as the mean value of a Gaussian fit of the reconstructed mass distribution of OS tau pairs in a MC sample of $Z \rightarrow \tau \tau$ events with associated jets.

## References

[1] Golfand YuA, Likhtman EP (1971). Extension of the algebra of poincare group generators and violation of p invariance. JETP Lett..

[2] Volkov DV, Akulov VP (1973). Is the neutrino a goldstone particle?. Phys. Lett. B.

[3] Wess J, Zumino B (1974). Supergauge transformations in four-dimensions. Nucl. Phys. B.

[4] Wess J, Zumino B (1974). Supergauge invariant extension of quantum electrodynamics. Nucl. Phys. B.

[5] Ferrara S, Zumino B (1974). Supergauge invariant Yang–Mills theories. Nucl. Phys. B.

[6] Salam A, Strathdee JA (1974). Supersymmetry and Nonabelian Gauges. Phys. Lett. B.

[7] Martin SP (1998). A Supersymmetry primer. Adv. Ser. Direct. High Energy Phys..

[8] Farrar GR, Fayet P (1978). Phenomenology of the production, decay, and detection of new Hadronic States associated with supersymmetry. Phys. Lett. B.

[9] Jungman G, Kamionkowski M, Griest K (1996). Supersymmetric dark matter. Phys. Rept..

[10] Goldberg H (1983). Constraint on the photino mass from cosmology. Phys. Rev. Lett..

[11] Ellis JR, Hagelin JS, Nanopoulos DV, Olive KA, Srednicki M (1984). Supersymmetric Relics from the big bang. Nucl. Phys. B.

[12] Evans L, Bryant P, Machine LHC (2008). JINST.

[13] Albornoz Vásquez D, Bélanger G, Bœhm C (2011). Revisiting light neutralino scenarios in the MSSM. Phys. Rev. D.

[14] Belanger G, Boudjema F, Cottrant A, Pukhov A, Semenov A (2005). WMAP constraints on SUGRA models with non-universal gaugino masses and prospects for direct detection. Nucl. Phys. B.

[15] King S, Roberts J, Roy D (2007). Natural dark matter in SUSY GUTs with non-universal gaugino masses. JHEP.

[16] Dine M, Fischler W (1982). A phenomenological model of particle physics based on supersymmetry. Phys. Lett. B.

[17] Alvarez-Gaume L, Claudson M, Wise MB (1982). Low-energy supersymmetry. Nucl. Phys. B.

[18] Nappi CR, Ovrut BA (1982). Supersymmetric extension of the SU(3) x SU(2) x U(1) model. Phys. Lett. B.

[19] Dine M, Nelson AE (1993). Dynamical supersymmetry breaking at low-energies. Phys. Rev. D.

[20] Dine M, Nelson AE, Shirman Y (1995). Low-energy dynamical supersymmetry breaking simplified. Phys. Rev. D.

[21] Dine M, Nelson AE, Nir Y, Shirman Y (1996). New tools for low-energy dynamical supersymmetry breaking. Phys. Rev. D.

[22] Randall L, Sundrum R (1999). Out of this world supersymmetry breaking. Nucl. Phys. B.

[23] Giudice GF, Luty MA, Murayama H, Rattazzi R (1998). Gaugino mass without singlets. JHEP.

[24] MSSM Working Group Collaboration, A. Djouadi et al., The minimal supersymmetric standard model (1998). arXiv:hep-ph/9901246

[25] Berger CF, Gainer JS, Hewett JL, Rizzo TG (2009). Supersymmetry without prejudice. JHEP.

[26] Alwall J, Le M-P, Lisanti M, Wacker JG (2008). Searching for directly decaying gluinos at the Tevatron. Phys. Lett. B.

[27] Alwall J, Schuster P, Toro N (2009). Simplified models for a first characterization of new physics at the LHC. Phys. Rev. D.

[28] LHC New Physics Working Group Collaboration, D. Alves, Simplified models for LHC New physics searches. J. Phys. G **39**, 105005 (2012). arXiv:1105.2838 [hep-ph]

[29] ATLAS Collaboration, Search for the direct production of charginos, neutralinos and staus in final states with at least two hadronically decaying taus and missing transverse momentum in $$pp$$ collisions at $$\sqrt{s} = 8\;\text{TeV}$$ with the ATLAS detector, JHEP **10**, 096 (2014). arXiv:1407.0350 [hep-ex]

[30] CMS Collaboration, Searches for electroweak production of charginos, neutralinos, and sleptons decaying to leptons and $$W$$, $$Z$$, and Higgs bosons in $$pp$$ collisions at $$8\;\text{ TeV }$$. Eur. Phys. J. C **74**, 3036 (2014). arXiv:1405.7570 [hep-ex] 10.1140/epjc/s10052-014-3036-7 PMC4370929 25814912

[31] CMS Collaboration, Search for supersymmetry in events with soft leptons, low jet multiplicity, and missing transverse energy in proton-proton collisions at $$\sqrt{s} = 8\;\text{ TeV }$$. Phys. Lett. B **759**, 9 (2016). arXiv:1512.08002 [hep-ex]

[32] The LEP SUSY Working Group and the ALEPH, DELPHI, L3 and OPAL experiments notes LEPSUSYWG/01-03.1, 04-01.1. http://lepsusy.web.cern.ch/lepsusy/Welcome.html

[33] ALEPH Collaboration, S. Schael, et al., Absolute mass lower limit for the lightest neutralino of the MSSM from $$e^+ e^-$$ data at $$\sqrt{s}$$ up to 209 GeV. Phys. Lett. B **583**, 247 (2004)

[34] DELPHI Collaboration, J. Abdallah et al., Searches for supersymmetric particles in e+ e- collisions up to 208 GeV and interpretation of the results within the MSSM. Eur. Phys. J. C **31**, 421 (2003)

[35] L3 Collaboration, M. Acciarri, et al., Search for charginos and neutralinos in $$e^{+} e^{-}$$ collisions at $$\sqrt{s}$$ = 189 GeV. Phys. Lett. B **472**, 420 (2000)

[36] OPAL Collaboration, G. Abbiendi et al., Search for chargino and neutralino production at $$\sqrt{s}$$ = 192 GeV to 209 GeV at LEP. Eur. Phys. J. C **35**, 1 (2004)

[37] ATLAS Collaboration, The ATLAS Experiment at the CERN Large Hadron Collider. JINST **3**, S08003 (2008)

[38] ATLAS Collaboration, ATLAS Insertable B-Layer Technical Design Report, ATLAS-TDR-19, 2010, http://cds.cern.ch/record/1291633, ATLAS Insertable B-Layer Technical Design Report Addendum, ATLAS-TDR-19-ADD-1 (2012). https://cds.cern.ch/record/1451888

[39] ATLAS Collaboration, Performance of the ATLAS trigger system. Eur. Phys. J. C **77**(2017), 317 (2015). arXiv:1611.09661 [hep-ex] 10.1140/epjc/s10052-017-4852-3 PMC5586243 28943784

[40] ATLAS Collaboration, Luminosity determination in $$pp$$ collisions at $$\sqrt{s} = 8\;\text{ TeV }$$ using the ATLAS detector at the LHC. Eur. Phys. J. C **76**, 653 (2016). arXiv:1608.03953 [hep-ex] 10.1140/epjc/s10052-016-4466-1 PMC5335615 28316496

[41] ATLAS Collaboration, The ATLAS simulation infrastructure. Eur. Phys. J. C **70**, 823 (2010). arXiv:1005.4568 [hep-ex]

[42] Agostinelli S (2003). GEANT4: a simulation toolkit. Nucl. Instrum. Methods A.

[43] ATLAS Collaboration, The simulation principle and performance of the ATLAS fast calorimeter simulation FastCaloSim, ATL-PHYS-PUB-2010-013 (2010). https://cds.cern.ch/record/1300517

[44] Sjöstrand T, Mrenna S, Skands PZ (2008). A brief introduction to PYTHIA 8.1. Comput. Phys. Commun..

[45] ATLAS Collaboration, Summary of ATLAS Pythia 8 tunes, ATL-PHYS-PUB-2012-003 (2012). https://cds.cern.ch/record/1474107

[46] Martin AD, Stirling WJ, Thorne RS, Wat G (2009). Parton distributions for the LHC. Eur. Phys. J. C.

[47] Gleisberg T (2009). Event generation with SHERPA 1.1. JHEP.

[48] Höche S, Krauss F, Schonherr M, Siegert F (2013). QCD matrix elements + parton showers: the NLO case. JHEP.

[49] Gleisberg T, Höche S (2008). Comix, a new matrix element generator. JHEP.

[50] Cascioli F, Maierhofer P, Pozzorini S (2012). Scattering amplitudes with open loops. Phys. Rev. Lett..

[51] Schumann S, Krauss F (2008). A Parton shower algorithm based on Catani–Seymour dipole factorisation. JHEP.

[52] Ball RD (2015). Parton distributions for the LHC Run II. JHEP.

[53] Catani S, Cieri L, Ferrera G, de Florian D, Grazzini M (2009). Vector boson production at hadron colliders: a fully exclusive QCD calculation at NNLO. Phys. Rev. Lett..

[54] Lai H-L (2010). New parton distributions for collider physics. Phys. Rev. D.

[55] ATLAS Collaboration, Multi-boson simulation for $$13\;\text{ TeV }$$ ATLAS analyses, ATL-PHYS-PUB-2016-002, 2016, https://cds.cern.ch/record/2119986

[56] Alioli S, Nason P, Oleari C, Re E (2010). A general framework for implementing NLO calculations in shower Monte Carlo programs: the POWHEG BOX. JHEP.

[57] Sjöstrand T, Mrenna S, Skands PZ (2006). PYTHIA 6.4 physics and manual. JHEP.

[58] Skands PZ (2010). Tuning Monte Carlo generators: the perugia tunes. Phys. Rev. D.

[59] Lange DJ (2001). The EvtGen particle decay simulation package. Nucl. Instrum. Methods A.

[60] Czakon M, Mitov A (2014). Top++: a program for the calculation of the top-pair cross-section at hadron colliders. Comput. Phys. Commun..

[61] Kidonakis N (2010). Two-loop soft anomalous dimensions for single top quark associated production with a W- or H-. Phys. Rev. D.

[62] Kant P (2015). HatHor for single top-quark production: updated predictions and uncertainty estimates for single top-quark production in hadronic collisions. Comput. Phys. Commun..

[63] Alwall J (2014). The automated computation of tree-level and next-to-leading order differential cross sections, and their matching to parton shower simulations. JHEP.

[64] ATLAS Collaboration, ATLAS Pythia 8 tunes to $$7\;\text{ TeV }$$ data, ATL-PHYS-PUB-2014-021, 2014. https://cds.cern.ch/record/1966419

[65] Ball RD (2013). Parton distributions with LHC data. Nucl. Phys. B.

[66] Lazopoulos A, McElmurry T, Melnikov K, Petriello F (2008). Next-to-leading order QCD corrections to $$t \bar{t} Z$$ production at the LHC. Phys. Lett. B.

[67] Campbell JM, Ellis RK (2012). $$t \bar{t} W^{+-}$$ production and decay at NLO. JHEP.

[68] Lönnblad L, Prestel S (2012). Matching tree-level matrix elements with interleaved showers. JHEP.

[69] Fuks B, Klasen M, Lamprea DR, Rothering M (2012). Gaugino production in proton–proton collisions at a center-of-mass energy of 8 TeV. JHEP.

[70] Fuks B, Klasen M, Lamprea DR, Rothering M (2013). Precision predictions for electroweak superpartner production at hadron colliders with Resummino. Eur. Phys. J. C.

[71] Borschensky C (2014). Squark and gluino production cross sections in pp collisions at $$\sqrt{s}$$ = 13, 14, 33 and 100 TeV. Eur. Phys. J. C.

[72] ATLAS Collaboration, Vertex Reconstruction Performance of the ATLAS Detector at$$\sqrt{s} = 13\;\text{ TeV }$$, ATL-PHYS-PUB-2015-026 (2015). https://cds.cern.ch/record/2037717

[73] ATLAS Collaboration, Topological cell clustering in the ATLAS calorimeters and its performance in LHC Run 1. arXiv:1603.02934 [hep-ex] 10.1140/epjc/s10052-017-5004-5 PMC5586976 28943797

[74] Cacciari M, Salam GP, Soyez G (2008). The anti-$$k_T$$ jet clustering algorithm. JHEP.

[75] Cacciari M, Salam GP, Soyez G (2012). FastJet user manual. Eur. Phys. J. C.

[76] ATLAS Collaboration, Jet energy measurement with the ATLAS detector in proton–proton collisions at $$\sqrt{s} = 7\;\text{ TeV }$$. Eur. Phys. J. C **73**, 2304 (2013). arXiv:1112.6426 [hep-ex]

[77] ATLAS Collaboration, Jet calibration and systematic uncertainties for jets reconstructed in the ATLAS Detector at $$\sqrt{s} = 13\;\text{ TeV }$$. ATL-PHYS-PUB-2015-015 (2015). https://cds.cern.ch/record/2037613

[78] Cacciari M, Salam GP (2008). Pileup subtraction using jet areas. Phys. Lett. B.

[79] ATLAS Collaboration, Tagging and suppression of pileup jets with the ATLAS detector, ATLAS-CONF-2014-018 (2014). https://cds.cern.ch/record/1700870

[80] ATLAS Collaboration, Performance of $$b$$-Jet identification in the ATLAS experiment. JINST **11**, P04008 (2016). arXiv:1512.01094 [hep-ex]

[81] ATLAS Collaboration, Expected performance of the ATLAS $$b$$-tagging algorithms in Run-2, ATL-PHYS-PUB-2015-022 (2015). https://cds.cern.ch/record/2037697

[82] ATLAS Collaboration, Optimisation of the ATLAS $$b$$-tagging performance for the 2016 LHC Run, ATL-PHYS-PUB-2016-012 (2016). https://cds.cern.ch/record/2160731

[83] ATLAS Collaboration, Electron efficiency measurements with the ATLAS detector using the 2015 LHC proton–proton collision data. ATLAS-CONF-2016-024 (2016). https://cds.cern.ch/record/2157687 10.1140/epjc/s10052-017-4756-2 PMC5434979 28579919

[84] ATLAS Collaboration, Muon reconstruction performance of the ATLAS detector in proton–proton collision data at $$\sqrt{s} = 13\;\text{ TeV }$$. Eur. Phys. J. C **76**, 292 (2016). arXiv:1603.05598 [hep-ex] 10.1140/epjc/s10052-016-4120-y PMC5321258 28280436

[85] ATLAS Collaboration, Reconstruction, energy calibration, and identification of hadronically decaying tau leptons in the ATLAS experiment for Run-2 of the LHC. ATL-PHYS-PUB-2015-045 (2015). https://atlas.web.cern.ch/Atlas/GROUPS/PHYSICS/PUBNOTES/ATL-PHYS-PUB-2015-045 10.1140/epjc/s10052-015-3500-z PMC4498687 26190938

[86] ATLAS Collaboration, Identification and energy calibration of hadronically decaying tau leptons with the ATLAS experiment in $$pp$$ collisions at $$\sqrt{s} = 8\;\text{ TeV }$$. Eur. Phys. J. C **75**, 303 (2015). arXiv:1412.7086 [hep-ex] 10.1140/epjc/s10052-015-3500-z PMC4498687 26190938

[87] ATLAS Collaboration, Expected performance of missing transverse momentum reconstruction for the ATLAS detector at $$\sqrt{s} = 13\;\text{ TeV }$$. ATL-PHYS-PUB-2015-023 (2015). https://cds.cern.ch/record/2037700

[88] ATLAS Collaboration, Performance of missing transverse momentum reconstruction with the ATLAS detector in the first proton–proton collisions at $$\sqrt{s} = 13\;\text{ TeV }$$. ATL-PHYS-PUB-2015-027 (2015). https://cds.cern.ch/record/2037904

[89] Lester CG, Summers DJ (1999). Measuring masses of semi-invisibly decaying particles pair produced at hadron colliders. Phys. Lett. B.

[90] Barr A, Lester C, Stephens P (2003). A variable for measuring masses at hadron colliders when missing energy is expected; $$m_{\text{ T2 }}$$: the truth behind the glamour. J. Phys. G.

[91] Cowan G, Cranmer K, Gross E, Vitells O (2011). Asymptotic formulae for likelihood-based tests of new physics. Eur. Phys. J. C.

[92] Tovey D (2008). On measuring the masses of pair-produced semi-invisibly decaying particles at hadron colliders. JHEP.

[93] Polesello G, Tovey D (2010). Supersymmetric particle mass measurement with the boost-corrected contransverse mass. JHEP.

[94] Baak M (2015). HistFitter software framework for statistical data analysis. Eur. Phys. J. C.

[95] ATLAS Collaboration, Measurement of the inelastic proton–proton cross section at $$\sqrt{s}$$ = 13 TeV with the ATLAS detector at the LHC. Phys. Rev. Lett. **117**, 182002 (2016). arXiv:1606.02625 [hep-ex] 10.1103/PhysRevLett.117.182002 27834993

[96] Read AL (2002). Presentation of search results: the CLs technique. J. Phys. G.

[97] Available at HEPDATA, https://www.hepdata.net/record/78377

[98] ATLAS Collaboration, ATLAS Computing Acknowledgements 2016–2017, ATL-GEN-PUB-2016-002, https://cds.cern.ch/record/2202407

